# Using GeoMx DSP Spatial Proteomics to Investigate Immune Infiltration of NOD Mouse Islet and Exocrine Compartments

**DOI:** 10.1007/s11307-024-01961-7

**Published:** 2024-11-18

**Authors:** Hasim Tekin, Claes Lindhardt, Julie Christine Antvorskov, Nicolai Schou Bager, Signe Regner Michaelsen, Aušrinė Areškevičiūtė, Jonas Pordel Vind, Bjarne Winther Kristensen, Knud Josefsen

**Affiliations:** 1 Department of Pathology, The Bartholin Institute, Copenhagen University Hospital, Copenhagen, Denmark; 2 Department of Clinical Research, Steno Diabetes Center Copenhagen, Translational Type 1 Diabetes Research, Herlev, Denmark; 3 Danish Reference Centre for Prion Disease, Department of Pathology, Copenhagen University Hospital, Copenhagen, Denmark; 4 Biotech Research and Innovation Center, University of Copenhagen, Copenhagen, Denmark

**Keywords:** NOD mice, Islets of Langerhans, Exocrine pancreas, Immune infiltration, Spatial proteomics, Type 1 diabetes, Beta cell dysfunction, Protein expression, Pancreas, Digital pathology

## Abstract

**Purpose:**

Type 1 Diabetes (T1D) pathogenesis involves immune cells infiltrating pancreatic Islets of Langerhans, leading to T cell activation, beta cell destruction, and impaired insulin production. However, infiltration has a heterogenic nature that isn’t described in detail, as not all islets are infiltrated. The aim of this study was to investigate if the observed heterogeneity is coupled to differences in immune and/or dysfunctional status of islets or exocrine cells, and if specific markers could elucidate mechanistic details of T1D pathogenesis.

**Procedures:**

The GeoMx platform was used to spatially quantify protein levels in pancreatic islets and exocrine tissue in Non-Obese Diabetic (NOD) mice. The protein panel included 17 immune activity markers and nine dysfunction markers. Immunohistochemical (IHC) staining and digital image analysis was used to analyze select marker proteins.

**Results:**

Use of the GeoMx platform to investigate T1D was shown to be possible, as Granzyme B protein levels were found to be lower in distal islet areas when compared to proximal areas. Smooth Muscle Actin protein levels were higher in exocrine areas proximal to immune-infiltrated islets, when compared to distally located exocrine areas. Findings from GeoMx were however not observed in IHC-stained sections.

**Conclusions:**

This study demonstrates that investigating T1D is possible with spatial proteomics, as the assays revealed presence of heterogenic islet areas in NOD mice, which may play a role in T1D progression and escape from immune recognition. This study highlights the potential of spatial technologies for elucidating T1D pathogenesis and future treatment strategies.

**Supplementary Information:**

The online version contains supplementary material available at 10.1007/s11307-024-01961-7.

## Introduction

Despite years of research, Type 1 diabetes (T1D) still affects millions of adults and children with no cure in sight. A 2021 modeling study found the number of humans worldwide suffering from T1D to be around 8.4 million [1]. A range of genetic and environmental factors driving pathogenesis have been described [2], which leads to activation of autoimmune T cells [3]. These then target and destroy insulin-producing pancreatic beta cells, thus leaving the patient without insulin and inducing clinical symptoms. A range of regulatory factors turning dysfunctional have been examined, such as exosome release from islet mesenchymal-like cells which activates autoreactive B and T cells [4], dysfunction of regulatory tRNA-fragments which affects immune and beta cell crosstalk [5], and insulin peptides themselves acting as both antigen and functional hormone [6].

Emerging research challenges the classical view of T1D pathogenesis, which suggests that immune-mediated islet invasion destroys beta cells, causing clinical T1D [3]. However, a larger-than-predicted number of beta cells, not destroyed by immune cells, is observed in humans even years after clinical T1D, reaching a “pseudo-atrophic” state whose mechanisms and reactivation potential remain unclear [7]. The antigen specificity of islet-infiltrating immune cells, which has been described for Non-obese diabetic mice [8], is a relevant clue, but antigen differences between attacked and surviving beta cells haven’t been described [3, 7, 9]. The relation between immune cells, beta cells, and infiltration remains poorly understood [10] and questioned, as recent observations propose that beta cells take an active role in promoting immune targeting [11]. There is thus a need to question how some beta cells avoid T cell targeting and if this is based on specific mechanisms, such as beta cells “hiding”, active/passive recruitment of T cells [12], novel antigens, or undiscovered mechanisms.

Comprising 90–99% of pancreatic mass, the exocrine compartment contains acinar cells essential for digestion [13]. Since total pancreatic mass is significantly decreased in patients with T1D compared to healthy controls, and islets only make up 1% of total mass, it is clear that T1D induces atrophy of the exocrine copmartment [3]. This is in part proposed to be a consequence of lowered insulin release in patients [14, 15]. Additionally, patients experience complications such as exocrine insufficiency [15], and children with T1D-autoantibodies develop exocrine complications upon progressing to clinical T1D [16]. Despite these observations, it is not known whether immune infiltration negatively affects exocrine cell function and health.

 The NanoString GeoMx^®^ Digital Spatial Profiler (GeoMx) allows for detection of protein or RNA in Formalin-Fixed Paraffin-Embedded tissue sections [17], which enables precise localization of protein/RNA targets with preserved tissue morphology. By using unique barcodes, the GeoMx can quantify hundreds of proteins or full mRNA transcriptomes. The spatial feature allows research of cellular relationships and morphological relevance of pathogenic mechanisms, such as infiltration or intrapancreatic signaling. GeoMx and similar spatial proteomics platforms are used in a range of fields [18] including pancreatic research [19].

We observed that infiltration of both the islets and exocrine tissue areas were not happening in a uniform matter. We therefore investigated how presence of infiltrating T cells induces immunogenic and/or apoptosis-related changes in islet cells proximal and distal to infiltrating T cells, whether T cell presence induces beta cell injury prior to T cell attack, and if specific beta cell signals attract T cells to islets. According to our hypothesis, there should be a heterogenic intra-islet difference in cellular dysfunction and death markers based on proximity to infiltrating immune cells, observed in both Islet of Langerhans and exocrine pancreatic cells. Is heterogeneity a result of incomplete immune destruction, or are cells actively suppressing function to avoid immune detection?

## Materials and Methods

### Animals

Initially, we aimed to use pancreata from Non-obese diabetic (NOD) mice with overt diabetes, defined as a blood sugar level > 12.0mmol/l. However, islets from these mice were too severely infiltrated by immune cells and thus unusable. We instead used mice with blood sugar levels < 12.0 mmol/L where islet morphology was preserved and available for sampling. All applicable institutional and national guidelines for the care and use of animals were followed. 17 female NOD mice were raised in a SPF animal facility and fed regular chow until 13 weeks of age, after which blood glucose levels were measured. 11 mice were sacrificed, and their pancreata processed into Formalin-fixed paraffin-embedded (FFPE) blocks with standard protocols (Suppl. Methods S1).

### GeoMx Sampling

FFPE sections were processed according to the NanoString GeoMx slide preparation manual [20] (protocol in Suppl. Methods S2). Our assay included the *Immune Cell Profiling Panel Mouse Protein Core* and *Cell Death Panel Mouse Protein Module* (Suppl. Table S1), anti-insulin-AlexaFluor488-antibody (1:2000, Thermo Fisher #53-9769-82) to visualize islets, and anti-Cluster of differentiation 3-AlexaFluor647-antibody (1:100, Bio-Rad #MCA1477A647) to visualize infiltrating CD3+ cells. Slides were scanned in FITC/525nm, Cy3/568nm, and Cy5/666nm channels. To optimize the available surface area on the four slides placed into the GeoMx, we embedded 11 pancreata into a single FFPE block. As tissue preparation induces artifacts, and islet distribution and quality vary, only eight mice could be sampled. In total, 57 ROIs for the islet analysis (19 ROIs of proximal, distal, and non-infiltrated control) and 36 ROIs for exocrine analysis (18 ROIs of areas proximal and distal to infiltrated islets) were sampled. For post-sampling quantification of counts, the nCounter manual was followed [21]. Ultimately, we measured 17 immune-related, nine cell death-related, and six control proteins in five morphologically divided areas of interest.

###  Immunohistochemistry and Image Analysis

FFPE sections from the same multi-tissue block used for GeoMx were immunohistochemically stained by use of standard protocols (Suppl. Methods S3). Our imaging analysis only included six mice from which we could sample at least three control and three sets of proximally/distally divided islets per mouse. Islets with severe infiltration or artifacts were excluded. We sampled 42 control and 51 proximal/distal islets for SMA analysis, and 40 control and 30 proximal/distal islets for GZMB analysis. For exocrine analysis, we sampled 26 endocrine and exocrine ROIs for GZMB, and 30 endo- and exocrine ROIs for SMA. ROIs were analyzed in the digital pathology software QuPath v0.5.0, where intensity features for ROIs were calculated by use of a color deconvolution algorithm that isolates IHC signals [22].

### Statistics and Visualization

GeoMx counts were filtered through a quality control script in the GeoMx Analysis Tool v3.0.0.113, and normalized by background correction to control proteins. This was chosen over housekeeping normalization as it improved signal-to-noise ratios and detection of low counts in our analysis. Counts were scaled to number of nuclei to account for varying cell numbers in ROIs. The difference in protein expression in two areas of interest were compared with paired t-tests with Benjamini-Hochberg-correction for multiple testing. Prior to statistical testing, a control islet ROI was removed as it didn’t pass quality control. Calculated mean intensity features for IHC were subjected to paired t-tests. The Principal Component Analysis (PCA) plot, heatmap, volcano plots and boxplots were generated using RStudio v1.4.17.17. For the PCA plot, we took data points within the 95% confidence interval for further analysis. Figures were compiled with GNU Image Manipulation Program v2.10.36. For all experiments, a p-value < 0.05 was considered statistically significant.

## Results

**Fig. 1:**
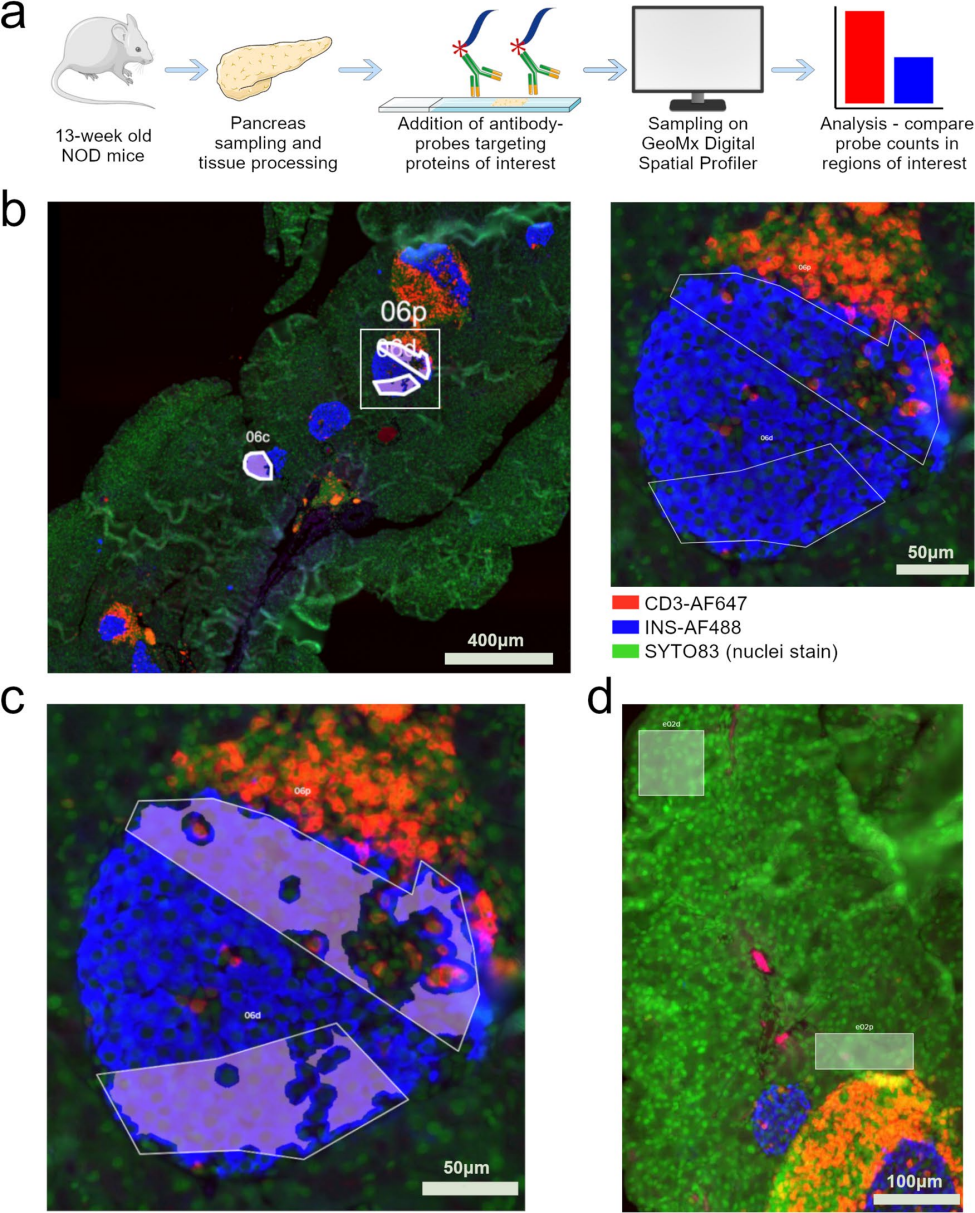
Sampling strategy for pancreatic regions of interest in NOD mice. **a** Schematic overview of assay. Pancreatic tissue from 13-week-old Non-obese diabetic (NOD) mice were processed and sectioned, and then incubated with probe-conjugated antibodies specific for proteins of interest. Sections were then placed in the GeoMx Digital Spatial Profiler where probes from regions of interest (ROI) can be collected for identification. Detection of a unique antibody-probe thus corresponds to detection of the protein recognized by the antibody. Probe counts are finally normalized and differences in probe levels in different ROIs compared statistically. **b** ROI sampling of Islets of Langerhans. Blue, anti-insulin-AlexaFluor488; red, anti-CD3-AlexaFluor647; green, SYTO83 nuclear stain. Left: overview of pancreatic tissue scanned with GeoMx. Scalebar 400 µm. Right: Zoom of squared area in the leftmost image. The areas within the islet (blue) are sampled proximal and distal to infiltrating CD3+ cells (red). Scalebar 50 µm**. c** A segmentation filter was added to the ROI which ensured that probes from red areas (CD3+ immune cells) were excluded during sample collection. Scalebar 50 µm. **d** Sampling strategy for exocrine pancreas. Areas of exocrine cells proximal and distal to islet immune infiltration were compared. Scalebar 100 µm

**Fig. 2:**
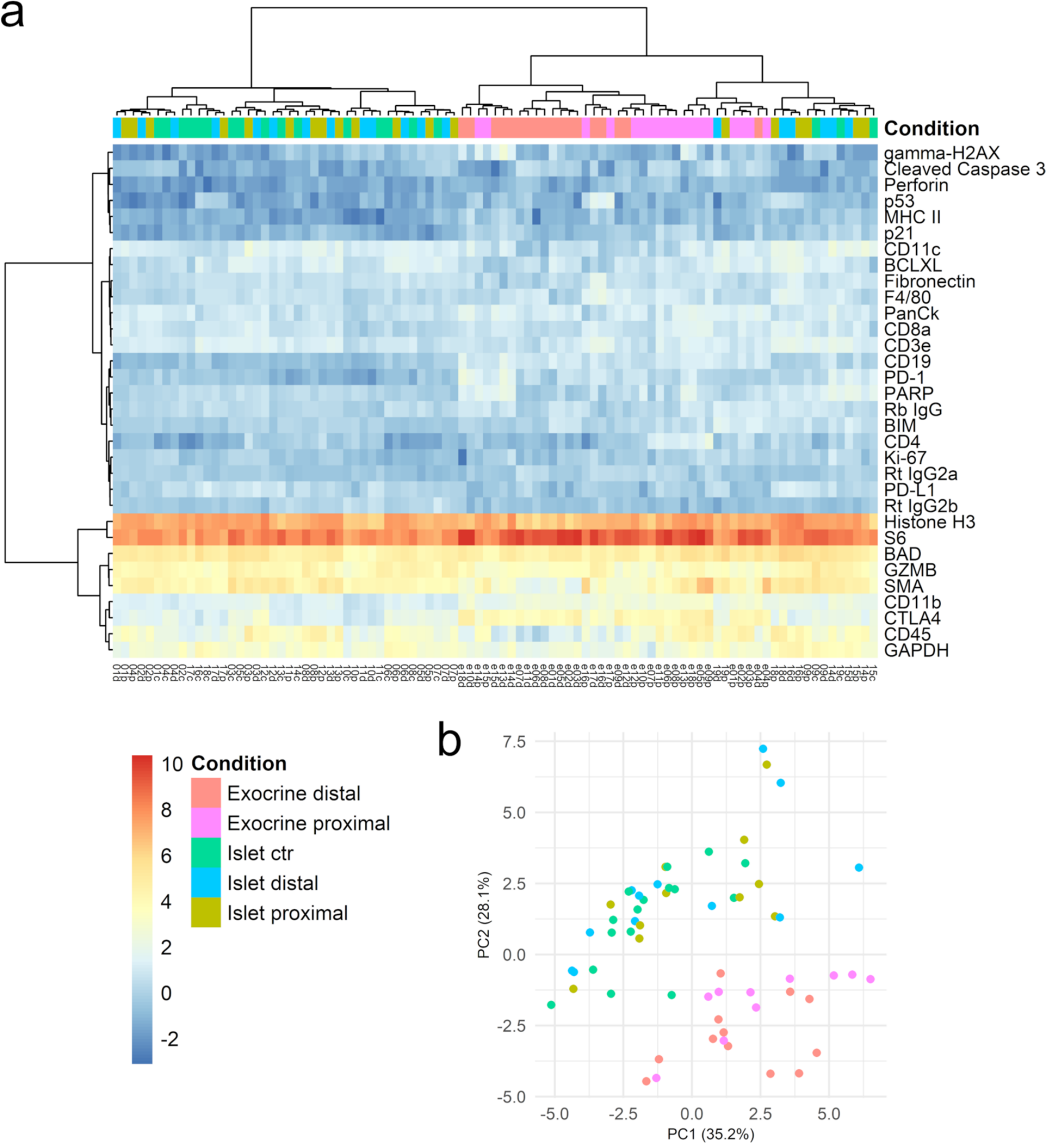
Global analysis of normalized GeoMx protein levels from sampled regions of interest. **a **Hierarchical cluster heatmap. Samples mainly cluster together based on their compartment within the islet i.e. being from an Islet of Langerhans or from exocrine tissue. **b** Principal component analysis. The highest two principal components (PCs) explain 63.3% of variation in the data. n=8

The GeoMx experiment and islet sampling strategy is summarized in Fig. 1a and b. Partially infiltrated islets were chosen, as they allowed proximal and distal sampling (Suppl. Fig. S1). Areas were drawn with adequate distance between proximal and distal Regions of interest (ROI), and a filter was added (Fig. 1c) to exclude CD3-positive signals within ROIs. This decreased the risk of mixing signals from invading cells with the signals from islets. For exocrine tissue, we similarly sampled areas close to and further away from infiltrated islets (Fig. 1d). Plotting normalized protein levels as a clustering heatmap (Fig. 2a) and performing a Principal Component Analysis (Fig. 2b) revealed that samples mainly cluster according to tissue type i.e., exocrine and endocrine compartments.

**Fig. 3:**
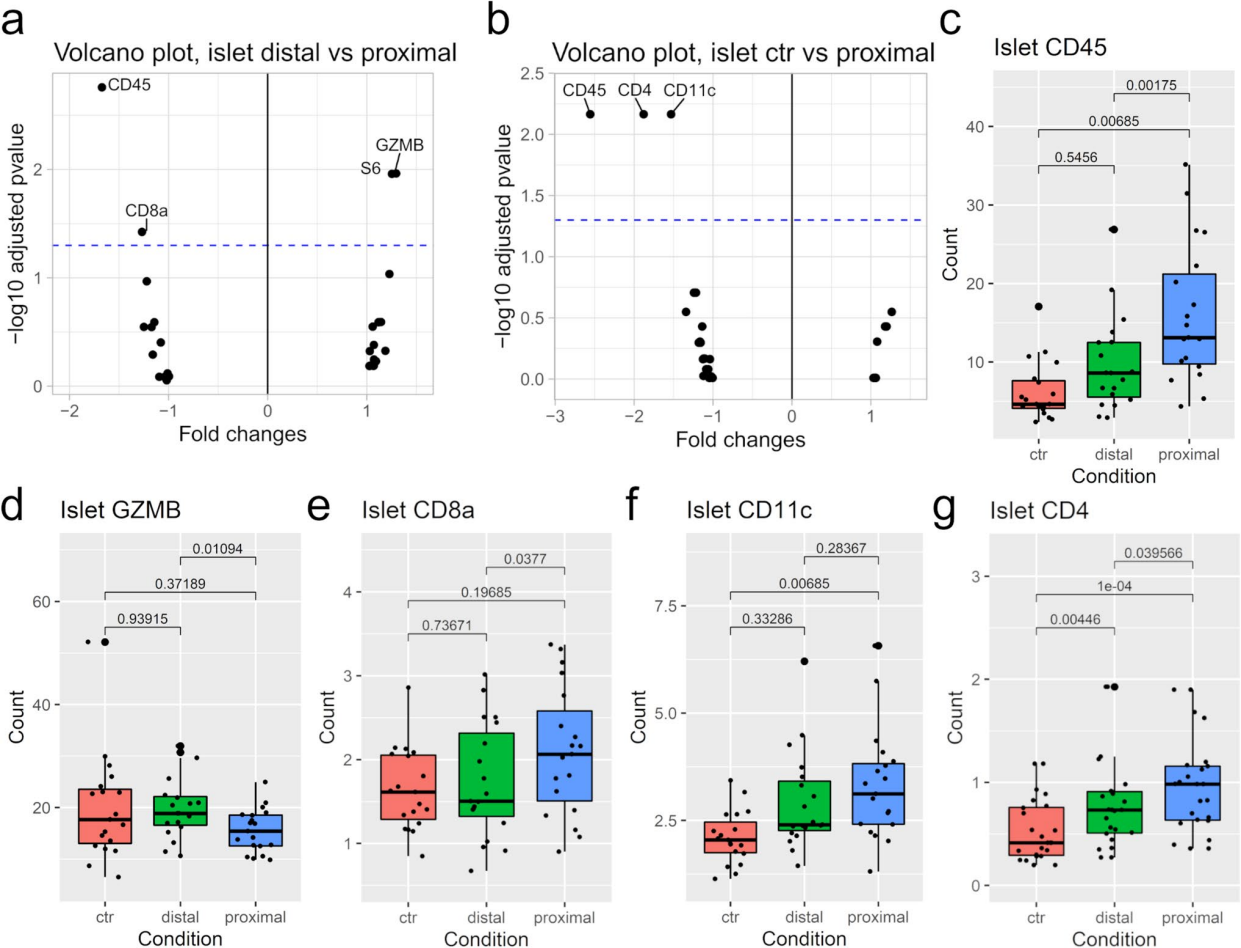
Protein expression in Islets of Langerhans. **a** Volcano plot comparing islet areas distal and proximal to infiltrating cells. A positive fold change indicates a higher protein level in distal areas, while a negative fold change indicates a higher protein level in proximal areas. The blue line indicates the 0.05 threshold for a significant corrected p-value. Abbreviations: CD45: Cluster of differentiation 45; CD8a: Cluster of differentiation 8 alpha; GZMB: Granzyme B; S6: Ribosomal protein S6. **b** Volcano plot comparing islet areas proximal to infiltration with non-infiltrated control islets. **c-g** Comparison of protein levels in all three conditions i.e. non-infiltrated control islets (ctr), islets distal to infiltration, and islets proximal to infiltration. Brackets show corrected p-values. Abbreviations: CD11c: Integrin alpha X; CD4: Cluster of differentiation 4; CD45: Cluster of differentiation 45.** c** CD4. **d** CD8a. **e** CD11c. **f** CD45. **g** GZMB. n=8

We compared relative protein levels in two islet conditions at a time for the 26 measured proteins. A negative fold change indicates that protein levels are higher in the comparison group (e.g., distal or proximal) relative to control; a positive fold change indicates that protein levels are higher in the control. When comparing islet areas distal and proximal to infiltration (Fig. 3a), protein levels of Cluster of differentiation 45 (CD45), Cluster of differentiation 8 alpha (CD8a), and Granzyme B (GZMB) were significantly altered: GZMB was higher in distal areas, while CD45 and CD8a were higher in proximal areas.

Next, comparing proximal islet areas to non-infiltrated control islets showed that proximal islet ROIs had significantly higher levels of CD45, Cluster of differentiation 4 (CD4), and Integrin alpha X (CD11c) (Fig. 3b). Compared to control islets, CD45 levels were higher in both proximal and distal islet ROIs (Fig. 3c), being highest in proximal areas. Thus, CD45 appeared to increase closer to infiltration. Since CD45 is minimally expressed in pancreatic cells [23, 24], these elevated levels may represent an artifact from infiltrating immune cells. No differences were observed in proteins when comparing proximal islet areas to control islets, thus revealing an interesting similarity despite presence/absence of nearby immune cells. No significant changes were observed for the remaining proteins (Suppl. Table S2, Suppl. Table S3).

GZMB levels were lower in proximal islet areas compared to the other areas (Fig. 3d). This is interesting, as this protein is present in NK and T cells and implicated in NK and T cell-mediated cell death [25]. The protein CD8a that is part of the cell surface CD8, was significantly higher in proximal areas compared to distal areas (Fig. 3e). However, CD8a levels of proximal control areas remained similar. As this protein is primarily found in T cells [26], this could be a measuring artifact. Compared to control islets, membrane protein CD11c levels were elevated in proximal islet areas, while levels were similar when comparing control and distal areas, and distal and proximal areas (Fig. 3f).

CD4 levels were lowest in control islets, then significantly higher in distal areas, and again significantly higher in proximal areas (Fig. 3g), i.e., higher levels closer to infiltration. Thus, this could be another measuring artifact. The protein S6 (Fig.  3a) is one of the three housekeeping proteins that is used to normalize protein levels. However, as we normalized by background subtraction, levels of S6 (Suppl. Fig. S2) and the two other housekeeping proteins Glyceraldehyde-3-phosphate dehydrogenase and Histone H3 could be ignored for downstream analysis.

**Fig. 4:**
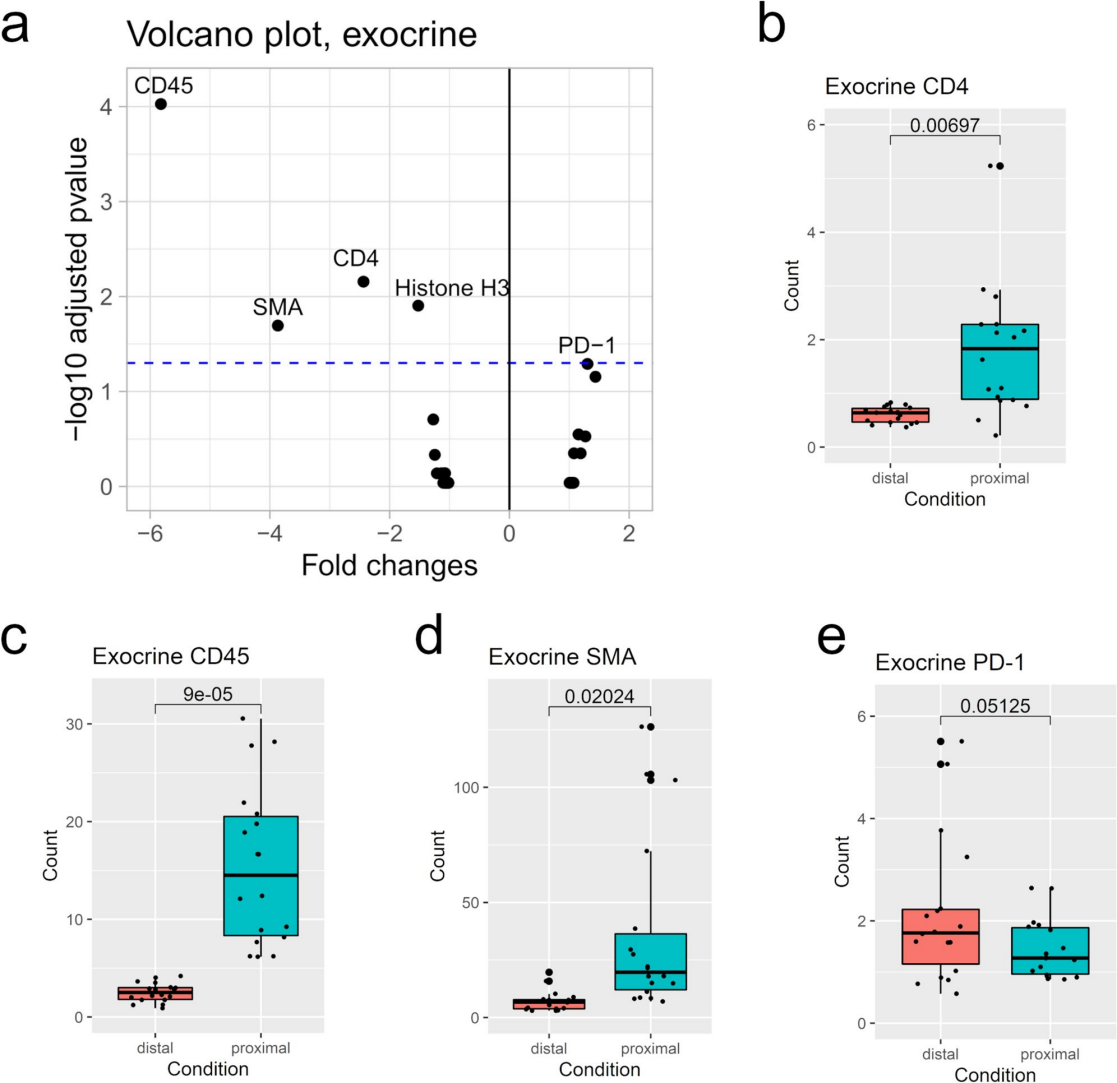
Protein expression in the exocrine pancreas. **a** Volcano plot comparing exocrine areas distal and proximal to an infiltrated islet of Langerhans. A positive fold change indicates a higher protein level in distal areas, while a negative fold change indicates a higher protein level in proximal areas. The blue dashed line indicates the 0.05 threshold for a significant corrected p-value. Note: PD-1 was only borderline significant (adj. p-value 0.0512). Abbreviations: CD4: Cluster of differentiation 4; CD45: Cluster of differentiation 45; PD-1: Programmed cell death protein 1, SMA: Smooth muscle actin.** b-e**: Individual comparison of protein levels in the two conditions. Brackets show corrected p-values. **b** CD4. **c** CD45. **d** SMA. **e** PD-1. n=8

After examining islets, we compared protein levels in exocrine pancreatic areas proximal and distal to infiltrated islets (Suppl. Table S4). Of the 26 measured proteins, CD45, CD4, and SMA levels were altered (Fig. 4a), Programmed cell death protein 1 (PD-1) levels borderline altered, and Histone H3 levels altered (Suppl. Fig. S2). Similar to islet findings, protein levels of CD4 (Fig. 4b) and CD45 (Fig. 4c) were significantly higher in exocrine tissue proximal to an islet infiltration, when compared to exocrine tissue distal to infiltration. SMA levels were also significantly higher in proximal areas compared to distal areas (Fig. 4d). PD-1 is a cell surface receptor expressed in T and B cells [27], whose levels here were decreased in proximal areas, but not enough to have a statistical relevance (Fig. 4e).

**Fig. 5:**
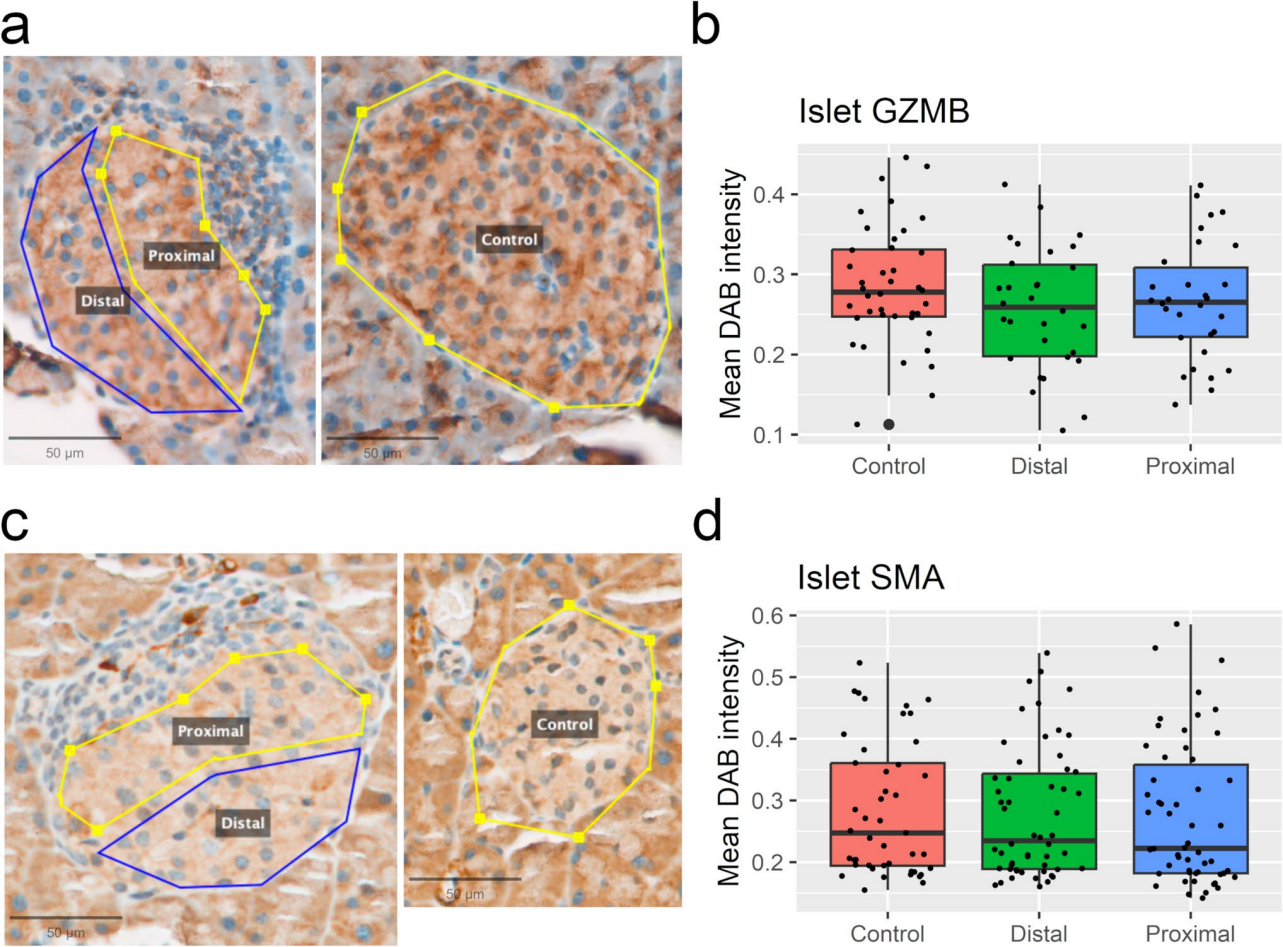
Immunohistochemical 3, 3'-diaminobenzidine (DAB) staining and analysis of mouse Islets of Langerhans. **a** Representative sampled Granzyme B (GZMB)-stained islets located proximal or distal to infiltrating immune cells (left), or non-infiltrated control islets (right). **b** Intensity levels of sampled islets. **c** Representative sampled Smooth muscle actin (SMA)-stained islets with conditions from (a). **d** SMA intensity levels. n=6. All scalebars 50 µm

**Fig. 6:**
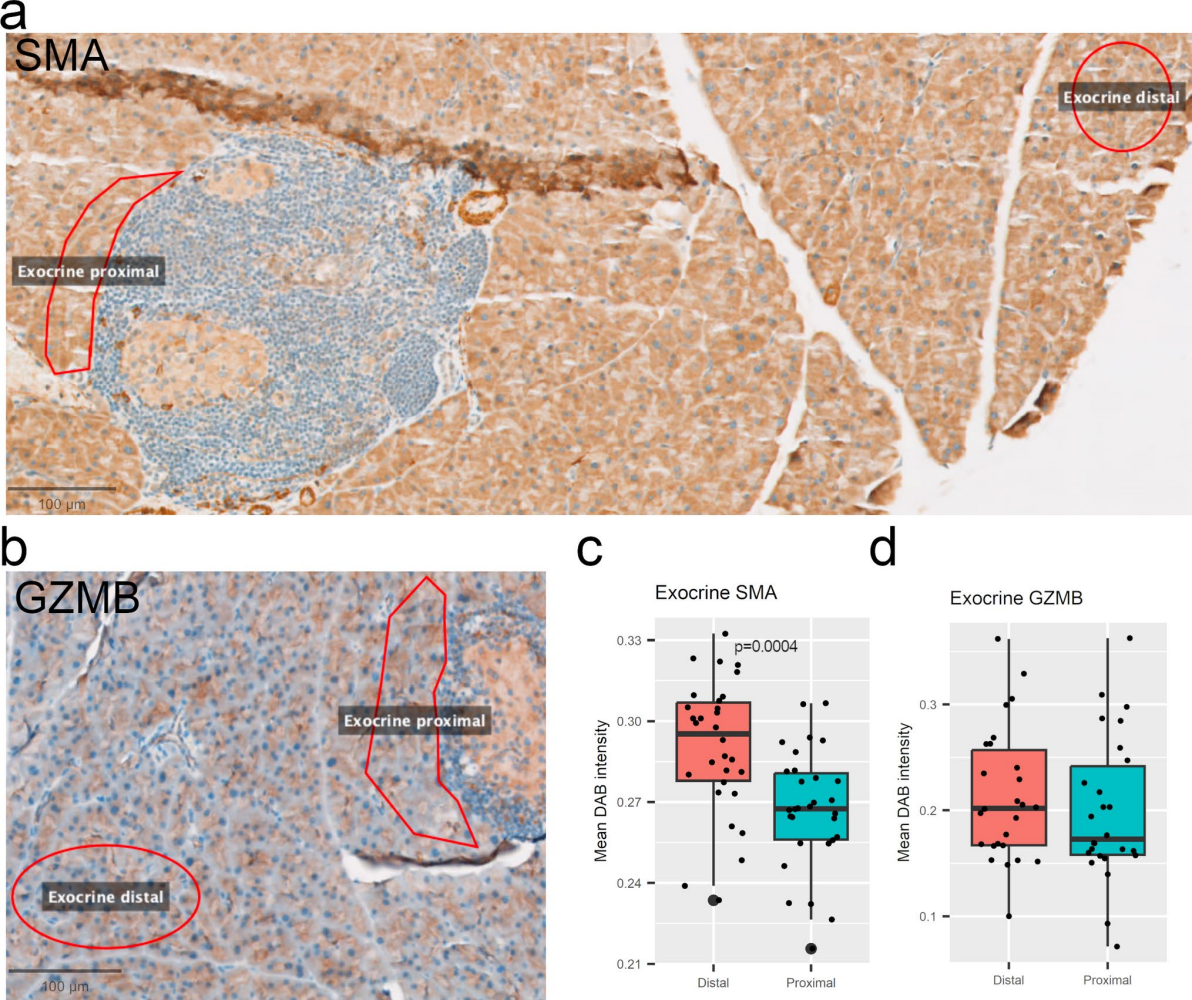
Immunohistochemical 3, 3'-diaminobenzidine (DAB) staining and analysis of mouse exocrine pancreatic tissue. **a** Representative sampled Smooth muscle actin (SMA)-stained islets located proximal or distal to infiltrated islets. Scalebar 100 µm. **b** Representative sampled Granzyme B (GZMB)-stained islets with conditions as in (a). Scalebar 400 µm. **c-d** Intensity levels of sampled islets. n=6

 To test whether the observed changes could be validated, we performed immunohistochemical (IHC) staining on the same pancreatic tissues. As GZMB levels were significantly altered in islets and SMA significantly altered in exocrine tissue, we chose these markers as candidates. We immunostained and used QuPath digital pathology software to analyze the proteins. Neither GZMB (Fig.  5a, b) nor SMA (Fig. 5c, d) protein staining intensities were altered when comparing proximal islet areas to distal islet areas. Protein levels in proximal or distal areas isolated were not altered when compared to control islets. For exocrine protein analysis, we sampled distal and proximal areas as for the GeoMx assay for SMA (Fig. 6a) and GZMB (Fig. 6b). In this protein intensity analysis, SMA levels (Fig. 6c) were significantly lower in proximal areas, albeit the difference being too small to be biologically relevant. No change in intensities were observed for GZMB (Fig. 6d).

## Discussion

The process of T cells invading and damaging the Islets of Langerhans during Type 1 Diabetes (T1D) development is complex and not fully understood, as it involves a range of pathways and mechanisms. The findings presented here challenge the traditional understanding of T1D pathogenesis in mice, shedding light on previously unexplored aspects of beta cell destruction and the interplay between pancreatic and immune cells. In this study, the quantification of 17 immune-related and nine cell death-related proteins across morphologically divided areas of interest have enriched understanding of T1D disease progression.

Our study found that protein levels of Integrin alpha X (CD11c), Cluster of differentiation 8 alpha (CD8a), Cluster of differentiation 45 (CD45), Granzyme B (GZMB), and Cluster of differentiation 4 (CD4) were significantly altered in Islets of Langerhans. Furthermore, CD4, CD45, and Smooth muscle actin (SMA) were significantly altered in exocrine pancreatic tissue proximal to infiltration.

We attempted to validate findings from the GeoMx Digital Spatial Profiler (GeoMx) with quantification of immunohistochemistry (IHC). We observed a difference in GZMB levels between proximal and distal islet sections using GeoMx, which could not be validated with IHC. This could be attributed to the higher sensitivity of the GeoMx or the sub-optimal performance of the antibody-IHC-QuPath analysis. Lastly, since the antibodies used in GeoMx and IHC most likely are of different clonalities, it is highly possible that these differences impact measured protein levels. We thus conclude that the IHC pipeline designed here could not validate findings from the GeoMx islet analysis. SMA levels in exocrine tissue were significantly altered in the IHC analysis, but the change wasn’t considered biologically relevant. We similarly observed that IHC analysis of exocrine GZMB did not confirm our GeoMx findings. Nevertheless, the observed differences in infiltrated islets raises interesting questions about the functional state of beta cells in T1D.

### Endocrine and Exocrine Findings

Elevated SMA levels are associated with Epithelial-to-mesenchymal transition (EMT) in cancerous tissue, where differentiated cells transition to mesenchymal states that facilitate invasion [28]. Additionally, induced dedifferentiation of mouse beta cells is known to trigger EMT [29]. In our case, SMA elevation could indicate that exocrine tissue proximal to infiltration is negatively affected by the islet infiltration, and thus uses EMT to protect itself from T cell-mediated detection and destruction. We did not detect alterations in islet SMA levels, and therefore cannot compare our results to detection of beta cell EMT in other studies [29]. There is a possibility that resolution of GeoMx cannot detect SMA changes in beta cells, as their protein levels could in theory be “masked” by non-altered SMA levels in remaining islet cells. This would result in the whole islet having unaltered SMA levels. Another explanation for observed exocrine differences could be a halo effect, where exocrine cells close to islets are affected by blood and other components leaking from islets to the outer environment [30]. Thus, observed differences in SMA levels could in theory be triggered by islet cells themselves, rather than by contact with infiltrating immune cells.

CD11c, a marker of dendritic cells in mice [31], was detected in islets with GeoMx but its presence is questionable as it is absent in beta cells in protein atlases [32]. Elevated CD8a and CD11c in proximal exocrine tissue was also detected. These most likely originate from resident lymphocytes within islets, as CD11c+, CD3+, and CD8+ cells are observed as islet resident cells [33, 34], or from externally infiltrating cells. Furthermore, CD11c+ cells are necessary for T cell trafficking to infiltrated islets in Non-obese diabetic (NOD) mice [35]. Together, these observations suggest that the detected CD11c signal is more likely to be from invading immune cells or from resident CD11c+ cells, than from endocrine islet cells.

Activated CD8+ T cells release perforin and GZMB upon reaching target cells [36], indicating that any GZMB detected in islet ROIs must be of T cell origin. Compared to CD11c or CD45, detection of GZMB in islet areas is reasonable since GZMB is not a receptor but a secreted enzyme. There is a possibility that immune cells are downregulating GZMB expression upon islet contact and killing beta cells by other mechanisms i.e. Fas-mediated apoptosis [9]. However, if this was true, we should have detected elevated levels of cleaved caspase 3, which we did not. Despite a likely T cell presence, we observe a decrease in GZMB and no change in perforin. This raises questions about the specific mechanisms employed by T cells to induce beta cell death, especially the observed imbalance in perforin vs. GZMB levels, since both are part of the same killing mechanism [37]. Interestingly, in islets we did not observe increased protein levels of the nine cell death markers in our panel, which suggests that islets were not in an elevated death state when infiltration began. As the control and infiltrated islets had similar levels of all cell death markers, we can speculate on whether islets were transitioning to a dysfunctional state even before immune infiltration.

CD4 is a T cell receptor coreceptor that assists in T cell activation [38]. Its presence in islet ROIs indicates that CD4+ T cells are contacting islets. Based on our results, is likely the same CD4+ T cell pools that are also present in exocrine areas. CD45 is described as a gatekeeper that prevents weak-binding antigens from activating T cell expansion and response [39]. It is possible that underlying mechanisms in individuals predisposed to T1D induces a malfunction, and autoantigens end up activating T cells instead of being inhibited by CD45 gatekeeping. The question of whether T cells are active at this spatial site (islet or exocrine proximal ROI) cannot be concluded here but warrants additional experiments, for example by sampling pancreatic macrophages which can present antigens to T cells [40, 41].

Findings of elevated CD45, CD4, CD11c, and CD8a strongly suggest that beta cell killing is driven by immune cells and inflammation [42, 43]. As CD45 and CD4 levels were altered in exocrine tissue, it is probable that islet and exocrine dysfunction is induced by the same pathogenic mechanisms. These observations fit with existing literature, but with this study we confirm that immune cell-mediated targeting of beta cells also takes place in heterogenic islet populations, and that both compartments of the pancreas are affected by T cell-driven immune activity.

## Limitations

The cluster of differentiation protein signals in beta cells are most likely a “bleed-through” of signals from nearby T cells, which occurred despite using filtering and a three µm border function of the GeoMx. This can be interpreted as a reflection of the sensitivity of the GeoMx, or a limitation which should be addressed. Other studies have validated that protein and RNA detected by GeoMx are similar to levels detected with IHC [44], thus serving as a partial validation. Since the fluorescent insulin stain could not be optimized for individual cells, the whole islet was stained and all cells within sampled. This led to inclusion of non-beta cells in the analysis, which most likely affected results negatively.

## Conclusions

We have demonstrated that GeoMx can be used to study T1D infiltration in endocrine and exocrine compartments. Future studies should investigate the molecular mechanisms underlying beta cell dysfunction in T1D, determine if specific signals from beta cells attract T cells to islets, and if pre-pathogenic T cell presence induces stress and damage in beta cells prior to T cell attack. Furthermore, understanding immune infiltration in humans and developing strategies to prevent or treat T1D would greatly benefit from spatial investigations of patient donor material. With these implementations, GeoMx and similar spatial platforms stand as valuable tools for future studies of T1D immune infiltration and pathogenesis.

## Supplementary Material

Below is the link to the electronic supplementary material.

Supplementary Material 1 (27.6 MB DOCX)

## Data Availability

Datasets and code for the manuscript can be found at our GitHub repository at https://github.com/claeslindhardt/IHCMousePancrea. Tissue slide scans can be found at https://dataverse.harvard.edu/dataverse/TekinetalSpatialNOD.
