# Supplementary material for: Using GeoMx DSP Spatial Proteomics to Investigate Immune Infiltration of NOD Mouse Islet and Exocrine Compartments
Source: Mol Imaging Biol. 2024 Nov 18;26(6):943–54. doi: 10.1007/s11307-024-01961-7 (PMC11634915; doi:10.1007/s11307-024-01961-7)
Supplement: Supplementary file 1 — Supplementary Material 1 (27.6 MB DOCX) [file 11307_2024_1961_MOESM1_ESM.docx]

**Supplementary Material**

Using GeoMx DSP Spatial Proteomics to investigate immune infiltration of NOD mouse islet and exocrine compartments

Hasim Tekin^1^, Claes Lindhardt^1^, Julie Christine Antvorskov^2^, Nicolai Schou Bager^1,4^, Signe Regner Michaelsen^1,4^, Aušrinė Areškevičiūtė^3^, Jonas Pordel Vind^1^, Bjarne Winther Kristensen^1,4^, and Knud Josefsen^1^

1. The Bartholin Institute, Copenhagen University Hospital, Copenhagen, Denmark.

2. Department of Clinical Research, Translational Type 1 Diabetes Research, Steno Diabetes Center Copenhagen, Herlev, Denmark.

3. Department of Pathology, Copenhagen University Hospital, Copenhagen, Denmark.

4. Biotech Research and Innovation Center, University of Copenhagen, Copenhagen, Denmark

Corresponding author:

Hasim Tekin

hasim.tekin@regionh.dk

+45 51 78 91 06

Suppl. Methods S1. Tissue processing procedures 3

Suppl. Methods S2. GeoMx protocol 3

Suppl. Methods S3. Immunohistochemical staining protocol 4

Suppl. Fig. S1. Sampled areas of interest from the GeoMx analysis 5

Suppl. Fig. S2. Boxplots for significantly altered housekeeping proteins 19

Suppl. Table S1. Proteins targeted by the Nano String Immune Cell Profiling Panel Mouse 20
Protein Core and the Cell Death Panel Mouse Protein Module.

Suppl. Table S2. Protein fold changes in islet areas of interest 21

Suppl. Table S3. Comparison of protein levels in islet distal and ctr areas of interest 23

Suppl. Table S4. Protein fold changes in exocrine areas of interest 25

Link to GitHub and Harvard Dataverse with additional material 27

Suppl. Methods S1. Tissue processing procedures

Processing of pancreas tissue to Formalin-fixed paraffin-embedded (FFPE) blocks. Dissected tissue sections were placed in 4% formaldehyde in phosphate-buffered saline (PBS). The following day, pancreata were embedded into paraffin with an automated tissue processor (Tissue-Tek VIP 6) by the following protocol: 4% formaldehyde in PBS 30 min; 70% ethanol 60 min; 96% ethanol 2x60 min; 99% ethanol 2x60 min followed by 1x90 min; Histo-Clear 3x60 min; paraffin 3x60 min followed by 1x90 min. All steps with solvents heated to 40 °C, except paraffin steps which were at 63 °C.

Suppl. Methods S2. GeoMx protocol

Adapted GeoMx protocol: prepared FFPE blocks were sectioned (3 µm) and placed onto TOMO adhesion slides (Matsunami Glass). Sections were deparaffinized with xylene for 3x5 min, then hydrated in 2x5 min of absolute ethanol, 2x5 min 95% ethanol, and 2x5 min ultrapure DEPC-treated water (Invitrogen #750023). Antigen retrieval was then performed by incubation in 1X citrate buffer (Sigma Aldrich #C9999) in 1X Tris-Buffered-Saline-Tween20 (TBS-T, Cell Signaling #9997S) in a pressure cooker (Crockpot #196494) for 15 min at 15 psi. Cooled slides were blocked in a humidity chamber for 1 hour at room temperature with GeoMx buffer W, followed by overnight 4°C incubation in antibody probe mix (NanoString Immune Cell Profiling Panel Mouse Protein Core and Cell Death Panel Mouse Protein Module) in buffer W. The detected proteins are listed in Suppl. Table S1. The probe mix also contained anti-insulin-AlexaFluor488-antibody (1:2000, Thermo Fisher #53-9769-82) to visualize islets, and anti-Cluster of differentiation 3-AlexaFluor647-antibody (1:100, Bio-Rad #MCA1477A647) to visualize infiltrating CD3+ cells. The following day, slides were washed with 1X TBS-T for 3x10 min, postfixed for 1 hour in 4% formaldehyde and 96% PBS, washed with 1X TBS-T 2x5 min, and incubated for 15 min with nuclear stain SYTO83 (1:1000, Molecular Probes #S-11364). Lastly, slides were washed in 1X TBS-T and placed in the GeoMx DSP. Slides were scanned in the FITC/525nm, Cy3/568nm, and Cy5/666nm channel, all with 50 ms exposure. ROIs were exposed to ultraviolet light to release and collect probes, which were stored at -80°C until used in nCounter analysis.

Suppl. Methods S3. Immunohistochemical staining protocol

Immunohistochemical (IHC) staining: FFPE sections from the same multi-tissue block used for GeoMx were mounted on TOMO slides and baked at 60°C for 30 minutes. Slides were then placed into the Ventana Discovery Ultra automated IHC/ISH instrument (Roche Diagnostics) and subjected to the following protocol (all reagents from Roche Diagnostics and room temperature unless specified): Deparaffinization at 70 °C 3x4 min; pretreatment with 100 °C CC1 buffer 32 min; DISCOVERY Inhibitor CM (#760-4307) 8 min; 100uL antibody diluted in 37 °C DISCOVERY Ab Diluent (#760-108) 32 min (Granzyme B 1:150 (GZMB, Invitrogen #PA5-96161); alpha-Smooth muscle actin 1:400 (SMA, Invitrogen)); secondary antibody DISCOVERY OmniMap anti-Rb HRP 20 min; DISCOVERY DAB CM, H2O2 CM, and Copper CM; Hematoxylin II 8 min; Bluing Reagent 4 min. Antibodies were validated in-house with positive (heart and spleen tissue), negative, and isotype (Negative Control Mouse IgG1 antibody) control stains. Stained slides were washed with soapy demineralized water until the Liquid Coverslip oil was washed off, and then dehydrated for 2 min each in the following ethanol concentrations: 70%, 96%, 99%, 99%, 99%, 99%. Lastly, coverslips were mounted with PERTEX mounting medium. Stained slides were scanned at 40x brightfield with the Vectra Polaris Automated Quantitative Pathology Imaging System (Akoya Biosciences, software v. 1.0.13).

Classifiations for QuPath analysis, i.e. how the digital image is processed and quantified, can be found in the online documentation for QuPath algorithm and analysis workflows, e.g. at the following link: https://qupath.readthedocs.io/en/0.4/docs/concepts/classifications.html.

Suppl. Fig. S1. Sampled areas of interest from the GeoMx analysis. Samples with suffix “p” denotes islet areas of interest proximal to immune cell infiltration; “d” islet areas distal to infiltration; “c” control non-infiltrated islets. Prefix “e” indicates a sample from exocrine areas, which are also divided into with proximal and distal areas with suffixes “p” and “d”, respectively. We initially used 17 female NOD mice, from which 11 mice were chosen after 13 weeks, as these fulfilled the cutoff of not being overt diabetic i.e. here interpreted as blood sugar levels <12 mmol/L, based on previous studies. Of these 11 mice, tissue sections were prepared for the GeoMx DSP. However, due to the stochastic nature of islet placement in tissues, we could only sample three unique islets for six of the 11 mice. Thus, the sample size for the GeoMx analysis is n=8, as we could sample at least one usable ROI from eight of the 11 mice. Four glass slides with tissue sections from these mice were sampled with the GeoMx DSP, and for each sampled ROI, the slide number (#1-4) is noted. If no control islet was present in the pancreas section from a given mouse, a control islet was sampled from a non-sampled mouse section. For two mice we could not find the necessary control islet: For mouse 6, we sampled one control islet from mouse 5 and one from mouse 4; For mouse 3, we sampled a control islet from mouse 5.

| Mouse #11. Slide #1. Conditions: Islet distal + proximal | Mouse #11. Slide #1. Condition: Control islet |
| --- | --- |
| 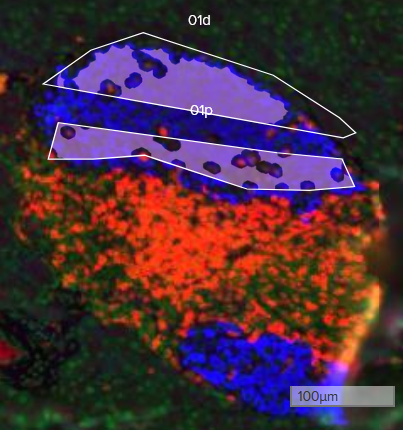 | 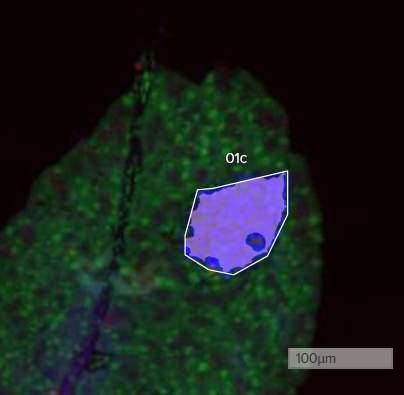 |
| Mouse #3. Slide #3.Conditions: Islet distal + proximal | Mouse #3. Slide #3. Condition: Control islet |
| 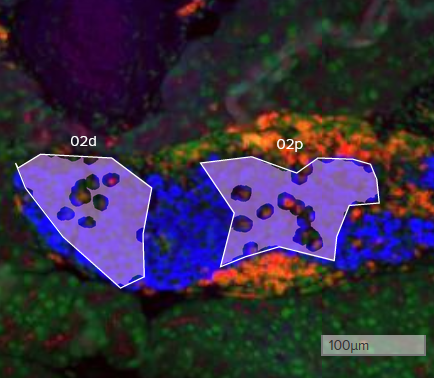 | 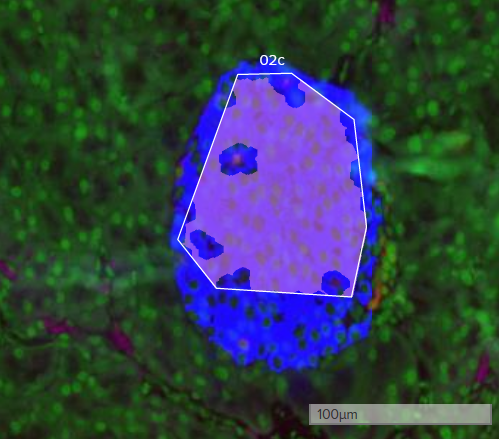 |
| Mouse #6. Slide #1. Conditions: Islet distal + proximal | Mouse #5. Slide #1. Condition: Control islet |
| 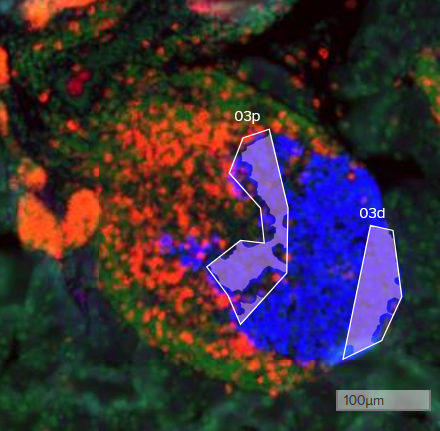 | 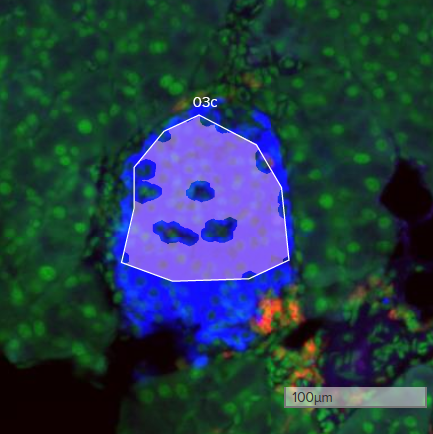 |
| Mouse #3. Slide #1. Conditions: Islet distal + proximal | Mouse #3. Slide #1. Condition: Control islet |
| 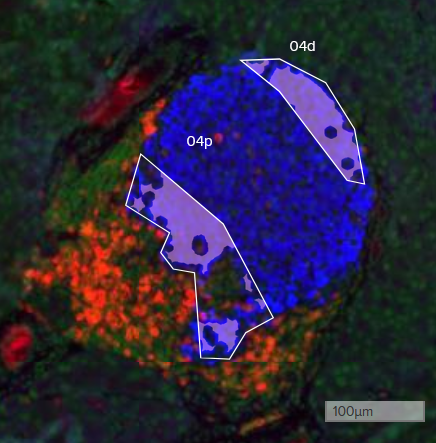 | 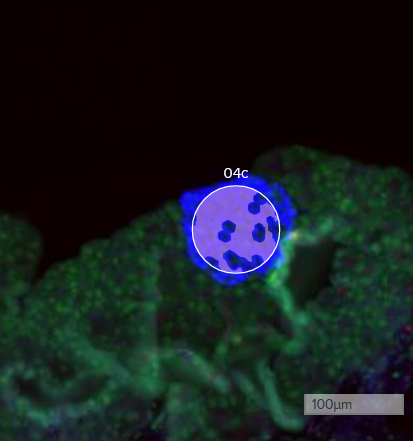 |
| Mouse #3. Slide #1. Conditions: Islet distal + proximal | Mouse #5. Slide #1. Condition: Control islet |
| 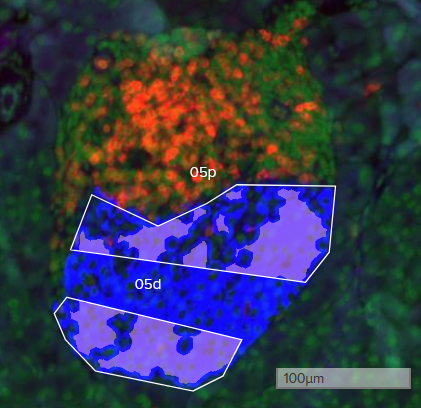 | 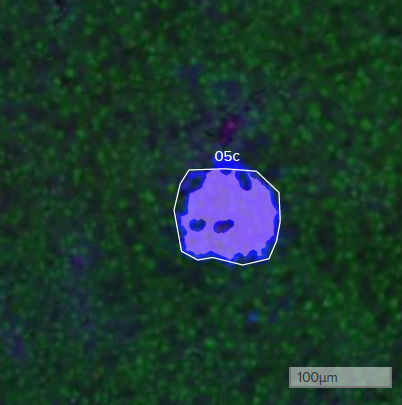 |
| Mouse #8. Slide #1. Conditions: Islet distal + proximal | Mouse #8. Slide #1. Condition: Control islet |
| 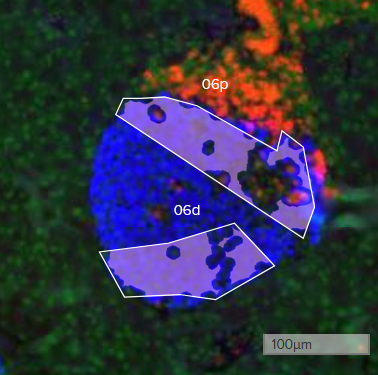 | 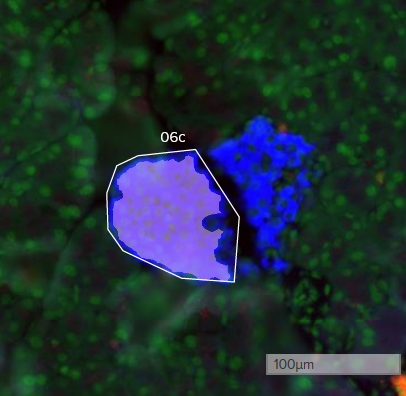 |
|  |  |
|  |  |
| Mouse #8. Slide #1. Conditions: Islet distal + proximal | Mouse #8. Slide #1. Condition: Control islet |
| 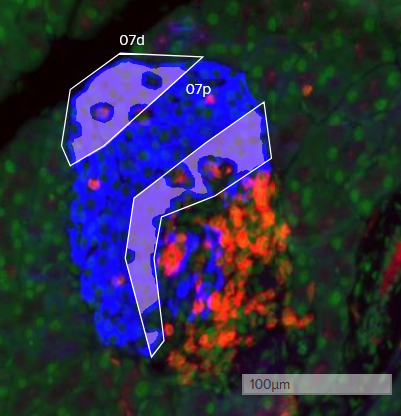 | 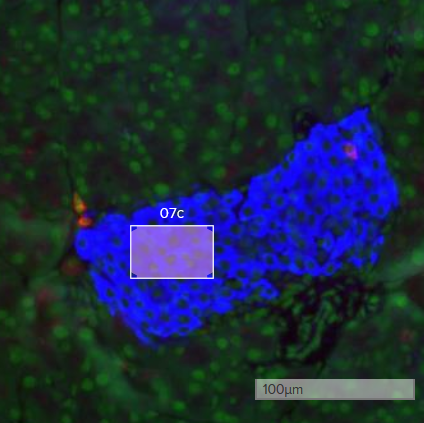 |
| Mouse #2. Slide #1. Conditions: Islet distal + proximal | Mouse #2. Slide #1. Condition: Control islet |
| 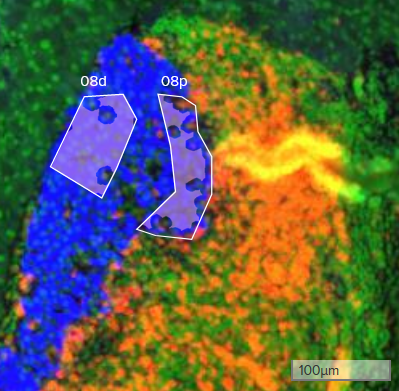 | 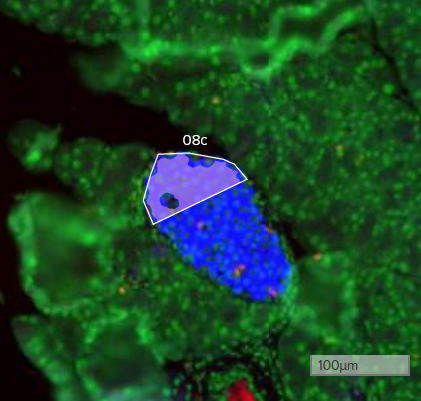 |
| Mouse #4. Slide #3. Conditions: Islet distal + proximal | Mouse #4. Slide #3. Condition: Control islet |
| 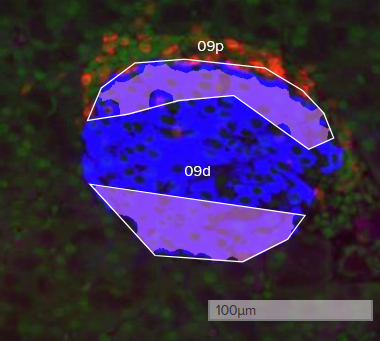 | 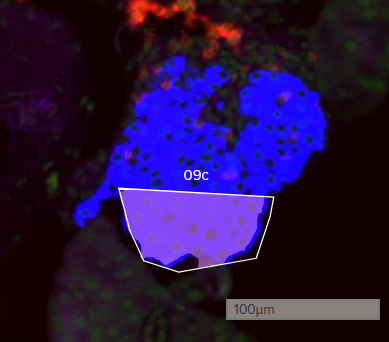 |
|  |  |
|  |  |
| Mouse #4. Slide #3. Conditions: Islet distal + proximal | Mouse #4. Slide #3. Condition: Control islet |
| 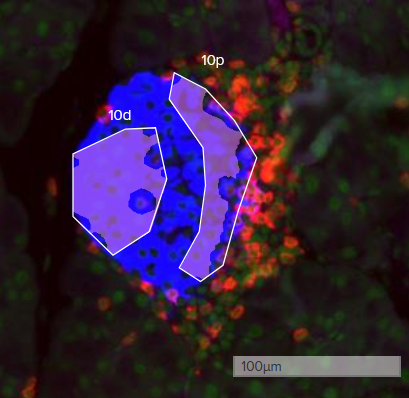 | 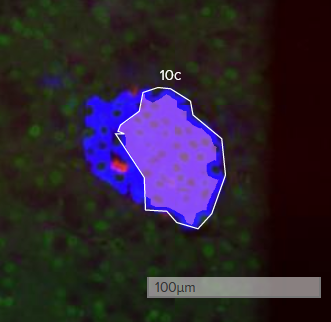 |
| Mouse 4#. Slide #3. Conditions: Islet distal + proximal | Mouse #4. Slide #3. Condition: Control islet |
| 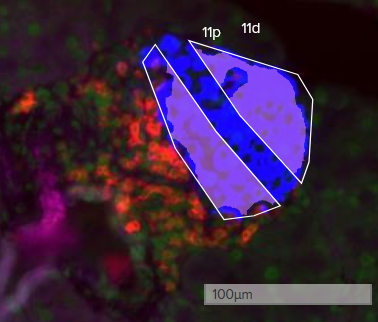 | 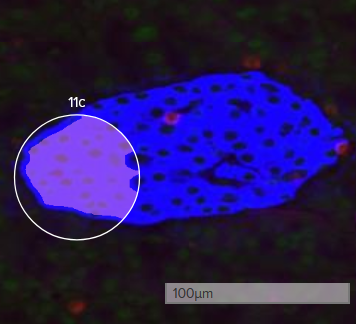 |
| Mouse #1. Slide #3. Conditions: Islet distal + proximal | Mouse #1. Slide #4. Condition: Control islet |
| 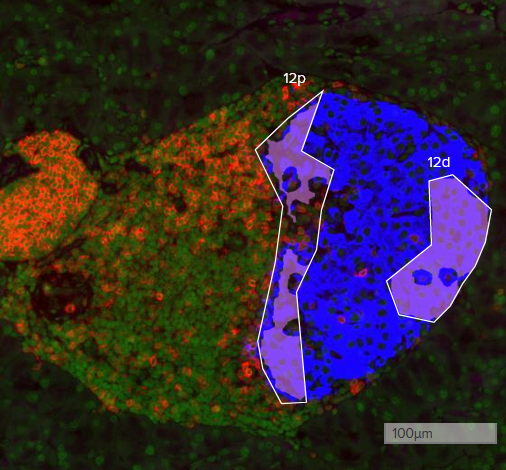 | 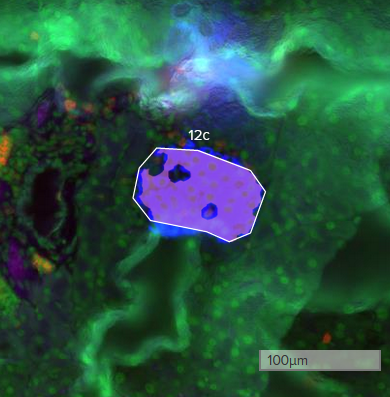 |
|  |  |
|  |  |
| Mouse #5. Slide #3. Conditions: Islet distal + proximal | Mouse #5. Slide #3. Condition: Control islet |
| 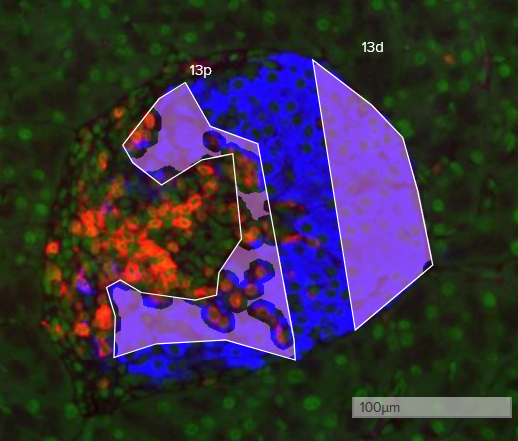 | 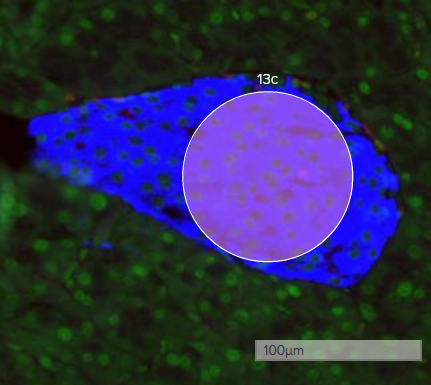 |
| Mouse #6 Slide #3. Conditions: Islet distal + proximal | Mouse #6. Slide #3. Condition: Control islet |
| 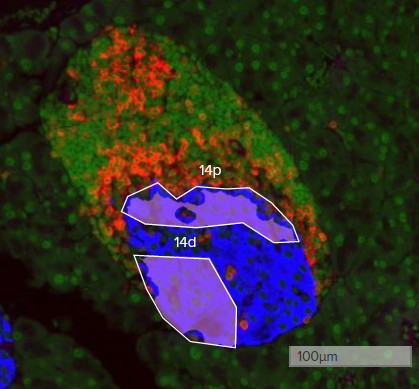 | 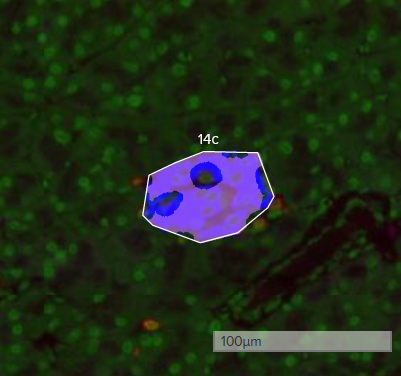 |
| Mouse #6. Slide #3. Conditions: Islet distal + proximal | Mouse #6. Slide #3. Condition: Control islet |
| 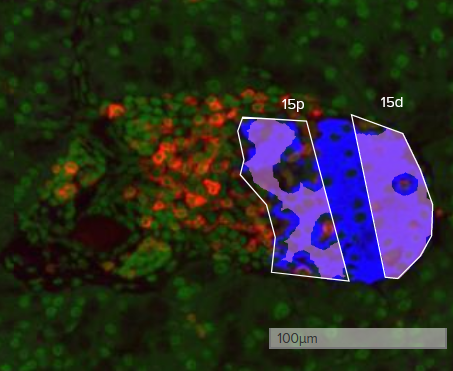 | 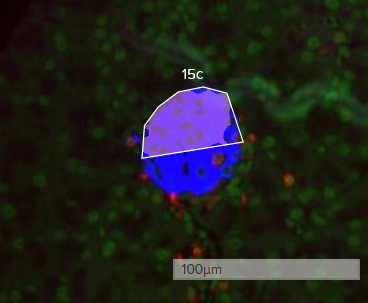 |
|  |  |
|  |  |
| Mouse #8. Slide #2. Conditions: Islet distal + proximal | Mouse #8. Slide #2. Condition: Control islet |
| 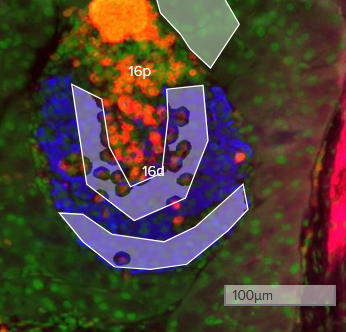 | 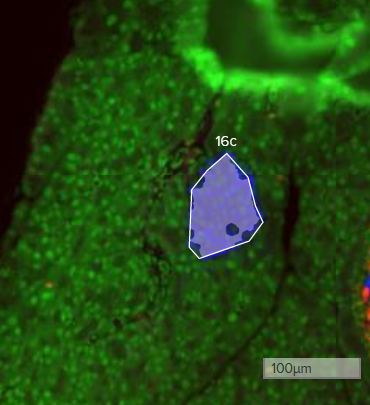 |
| Mouse #3. Slide #2. Conditions: Islet distal + proximal | Mouse #3. Slide #4. Condition: Control islet |
| 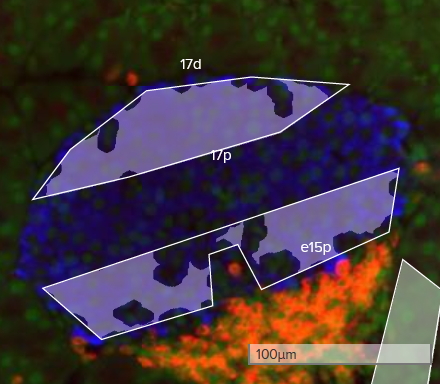 | 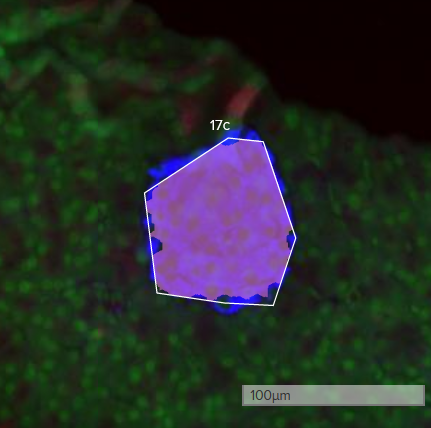 |
| Mouse #6. Slide #2. Conditions: Islet distal + proximal | Mouse #6. Slide #2. Condition: Control islet |
| 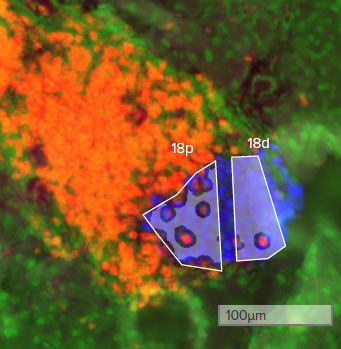 | 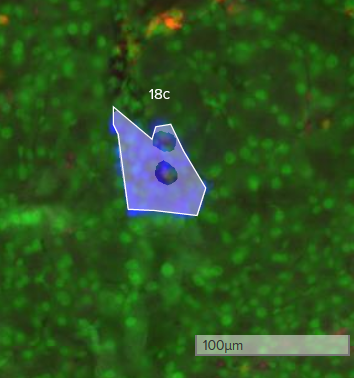 |
|  |  |
|  |  |
| Mouse #6. Slide #2. Conditions: Islet distal + proximal | Mouse #4. Slide #3. Condition: Control islet |
| 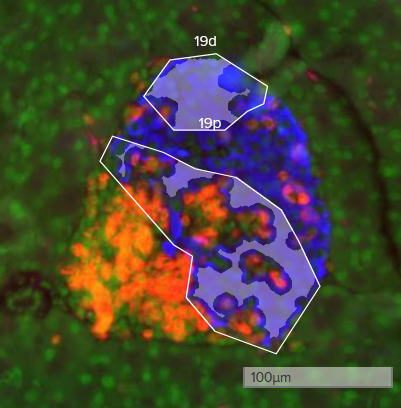 | 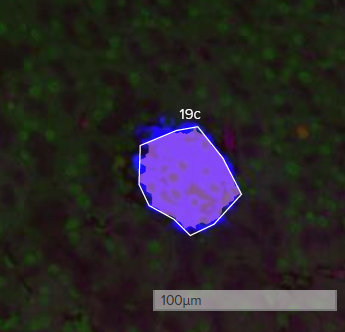 |
| Mouse #6. Slide #2. Condition: Exocrine proximal | Mouse #6. Slide #2. Condition: Exocrine distal |
| 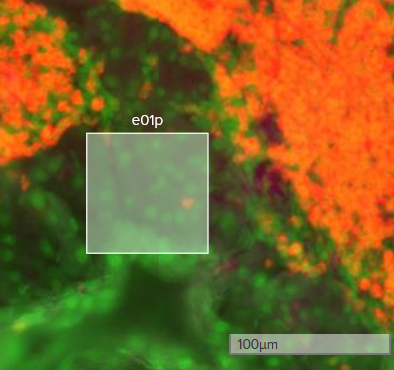 | 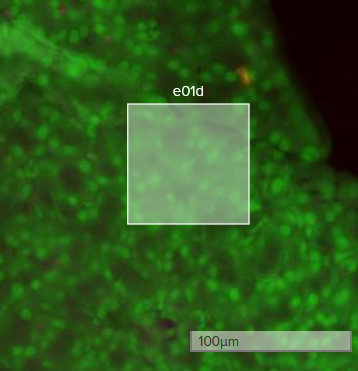 |
| Mouse #6. Slide #2. Condition: Exocrine proximal | Mouse #6. Slide #2. Condition: Exocrine distal |
| 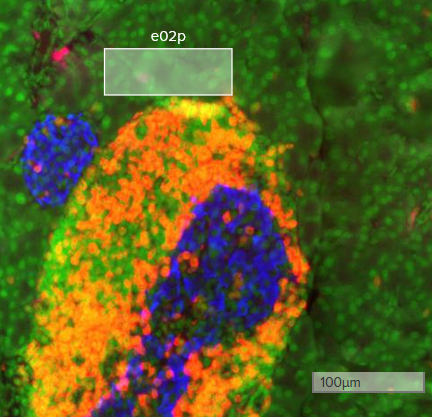 | 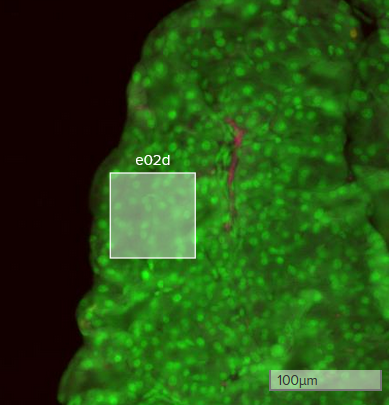 |

| Mouse #6. Slide #2. Condition: Exocrine proximal | Mouse #6. Slide #2. Condition: Exocrine distal |
| --- | --- |
| 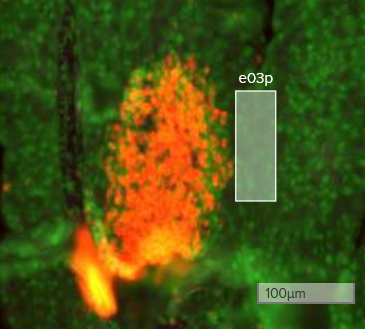 | 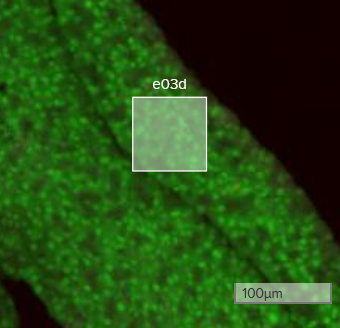 |
| Mouse #5. Slide #2. Condition: Exocrine proximal | Mouse #5. Slide #2. Condition: Exocrine distal |
| 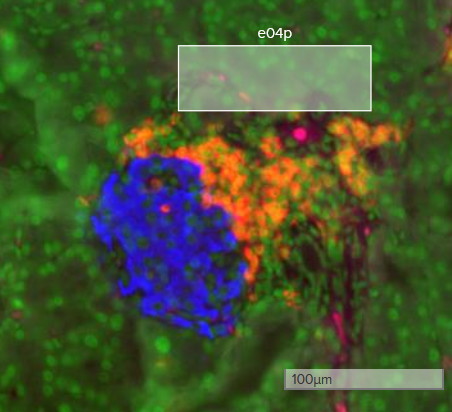 | 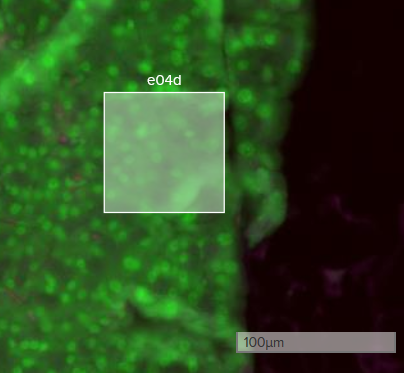 |
| Mouse #2. Slide #2. Condition: Exocrine proximal | Mouse #2. Slide #2. Condition: Exocrine distal |
| 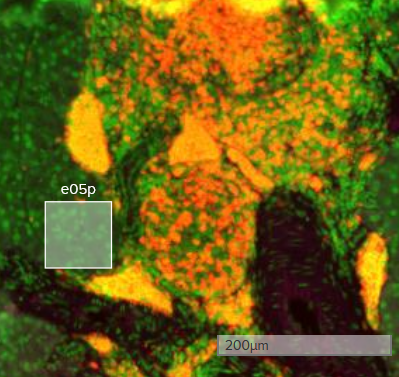 | 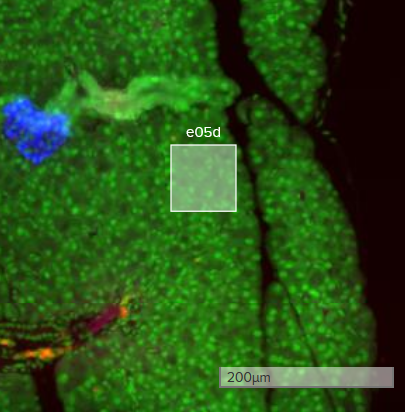 |

| Mouse #2. Slide #2. Condition: Exocrine proximal | Mouse #2. Slide #2. Condition: Exocrine distal |
| --- | --- |
| 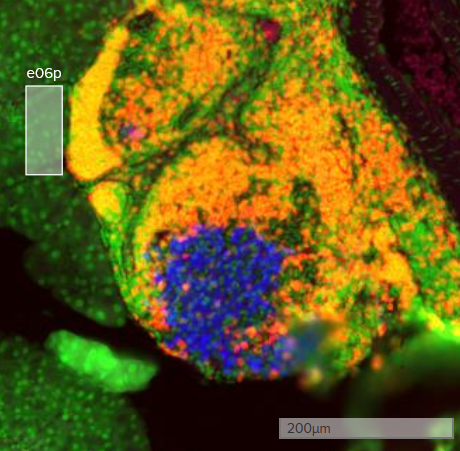 | 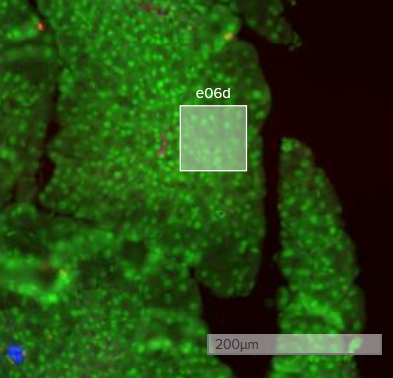 |
| Mouse #4. Slide #2. Condition: Exocrine proximal | Mouse #4. Slide #2. Condition: Exocrine distal |
| 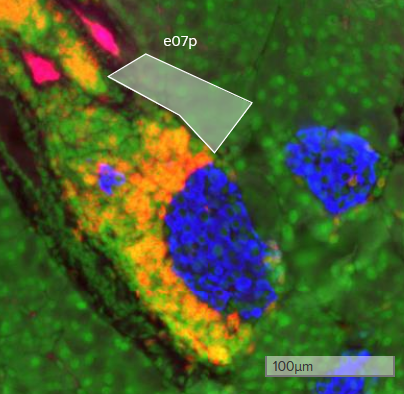 | 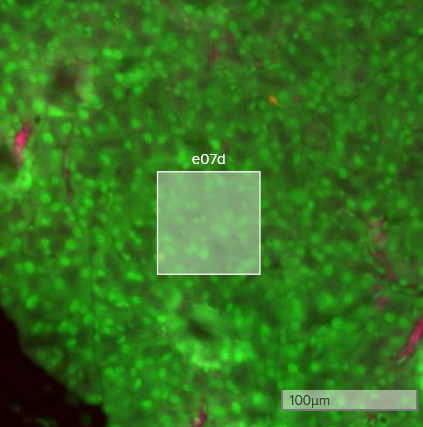 |
| Mouse #1. Slide #2. Condition: Exocrine proximal | Mouse #1. Slide #2. Condition: Exocrine distal |
| 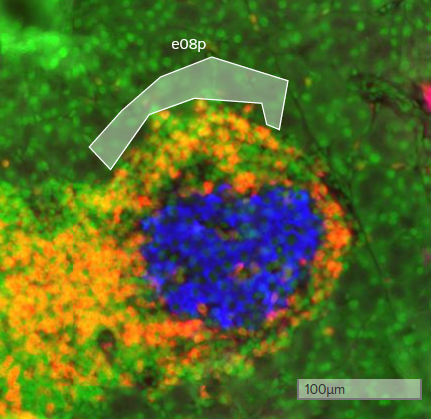 | 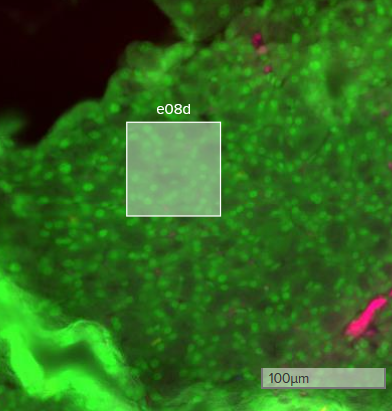 |
| Mouse #1. Slide #2. Condition: Exocrine proximal | Mouse #1. Slide #2. Condition: Exocrine distal |
| 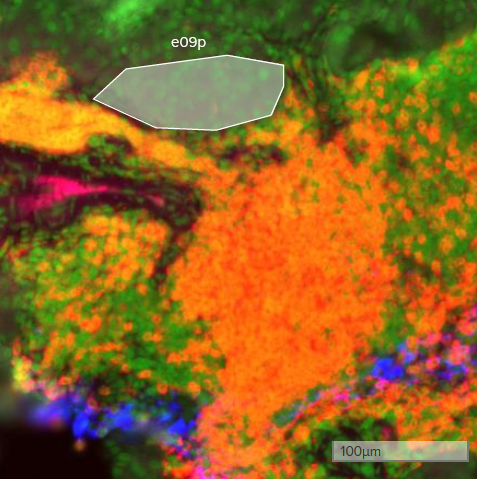 | 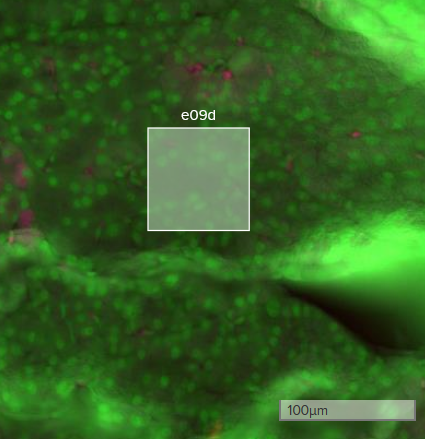 |
| Mouse #1. Slide #3. Condition: Exocrine proximal | Mouse #1. Slide #3. Condition: Exocrine distal |
| 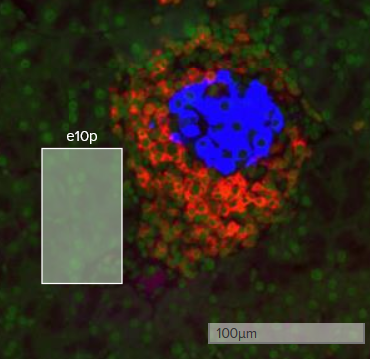 | 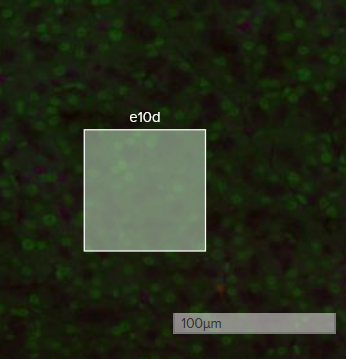 |
| Mouse #8. Slide #2. Condition: Exocrine proximal | Mouse #8. Slide #2. Condition: Exocrine distal |
| 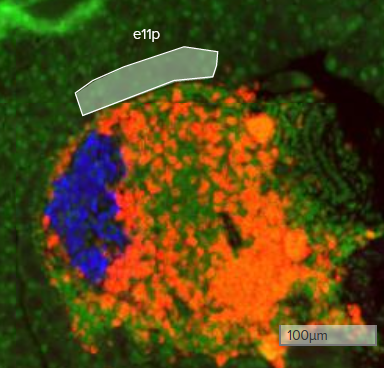 | 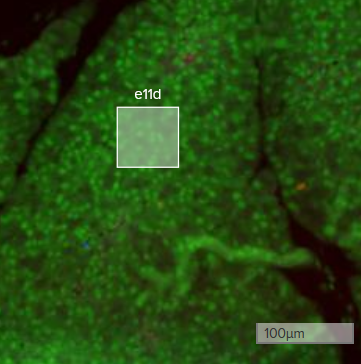 |
|  |  |
|  |  |
| Mouse #8. Slide #2. Condition: Exocrine proximal | Mouse #8. Slide #2. Condition: Exocrine distal |
| 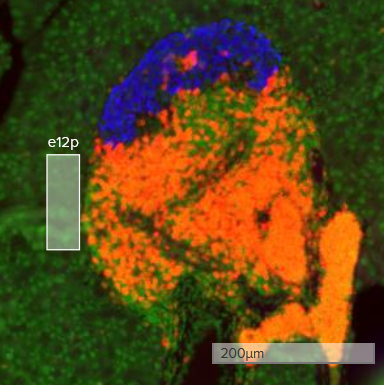 | 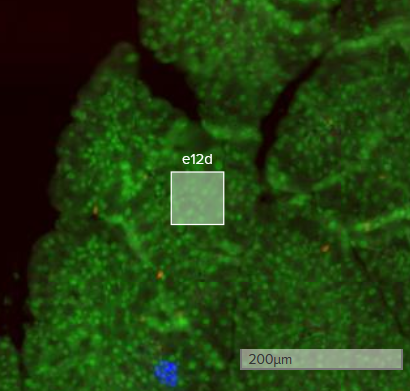 |
| Mouse #8. Slide #2. Condition: Exocrine proximal | Mouse #8. Slide #2. Condition: Exocrine distal |
| 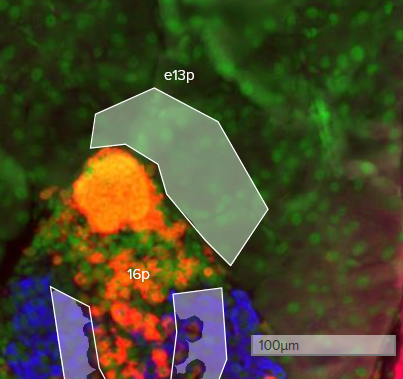 | 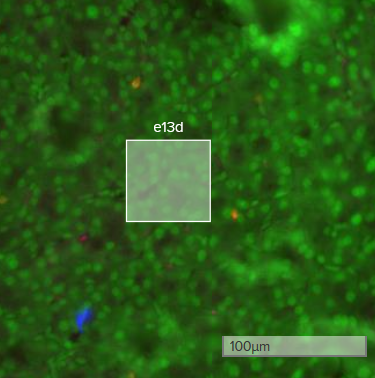 |
| Mouse #5. Slide #2. Condition: Exocrine proximal | Mouse #5. Slide #2. Condition: Exocrine distal |
| 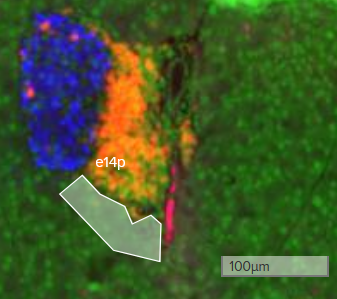 | 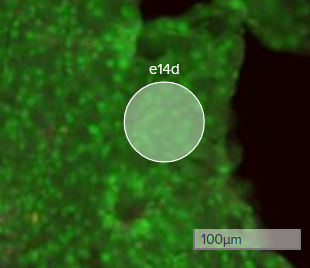 |

| Mouse #3. Slide #2. Condition: Exocrine proximal | Mouse #3. Slide #2. Condition: Exocrine distal |
| --- | --- |
| 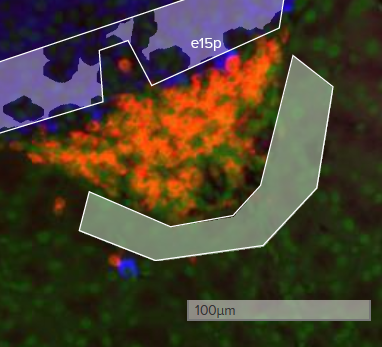 | 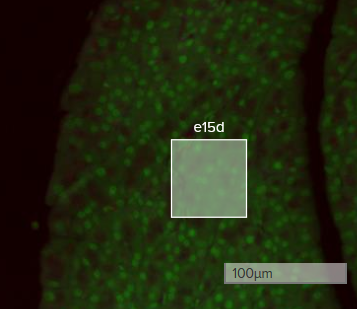 |
| Mouse #11. Slide #1. Condition: Exocrine proximal | Mouse #11. Slide #1. Condition: Exocrine distal |
| 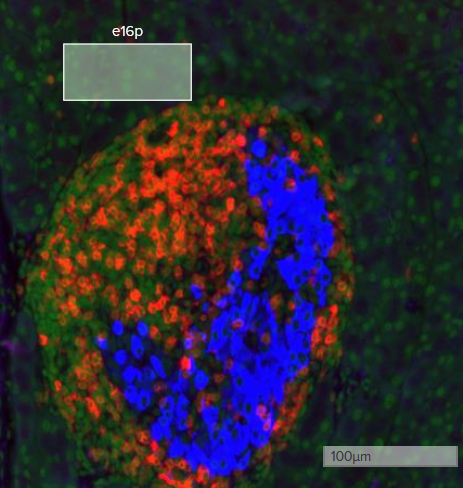 | 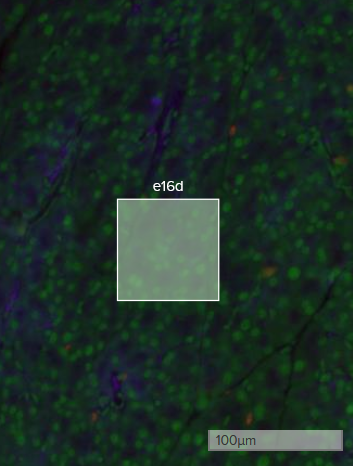 |
| Mouse #11. Slide #1. Condition: Exocrine proximal | Mouse #11. Slide #1. Condition: Exocrine distal |
| 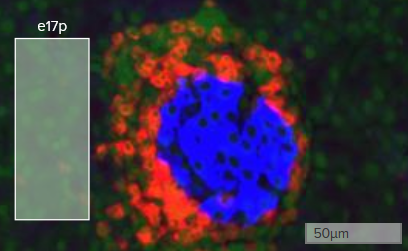 | 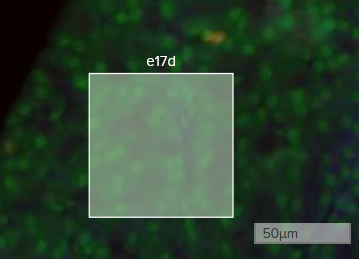 |

| Mouse #5. Slide #2. Condition: Exocrine proximal | Mouse #5. Slide #2. Condition: Exocrine distal |
| --- | --- |
| 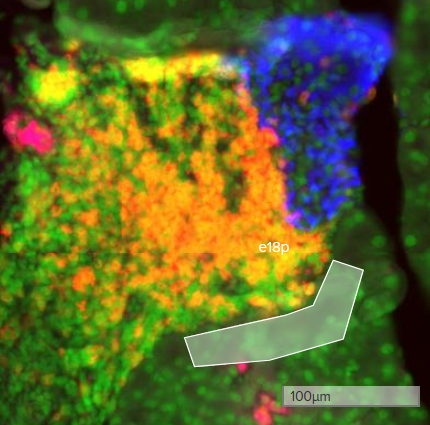 | 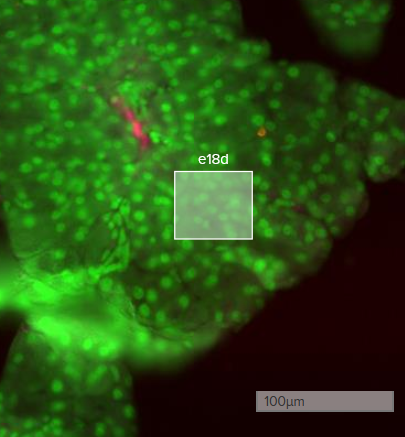 |

Suppl. Fig. S2. Boxplots for significantly altered housekeeping proteins. As background correction is used for this dataset, the housekeeping protein levels can be quantitatively determined. **(a)** Measured islet protein levels for housekeeping protein Ribosomal protein S6. **(b)** Measured protein levels for housekeeping gene Histone H3 in exocrine areas.


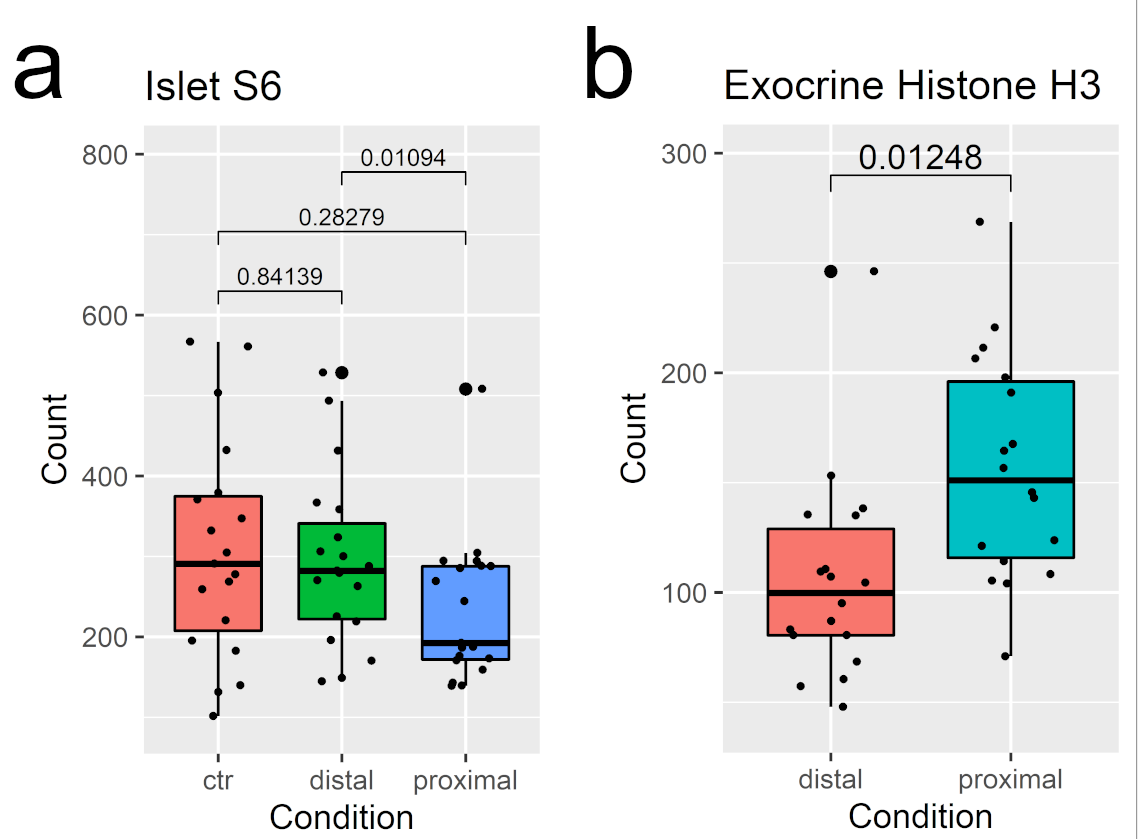


Suppl. Table S1. Proteins targeted by the Nano String Immune Cell Profiling Panel Mouse Protein Core and the Cell Death Panel Mouse Protein Module.

| Proteins detected by the Immune Cell Profiling Panel^1^ | Proteins detected by the Cell Death Panel | Control proteins detected by the Immune Cell Profiling Panel |
| --- | --- | --- |
| CD3e | BAD | GAPDH |
| CD4 | BCLXL | Histone H3 |
| CD8a | BIM | S6 |
| CD11b | Cleaved caspase 3 | Rabbit IgG^2^ |
| CD11c | gamma-H2AX | Rat IgG2a^2^ |
| CD19 | p21 | Rat IgG2b^2^ |
| CD45 | p53 |  |
| CTLA4 | PARP |  |
| Fibronectin | Perforin |  |
| F4/80 |  |  |
| GZMB |  |  |
| Ki-67 |  |  |
| MHC II |  |  |
| PanCk |  |  |
| PD-1 |  |  |
| PD-L1 |  |  |
| SMA |  |  |

^1^Abbreviations: BAD: BCL2-associated agonist of cell death; BCLXL: B-cell lymphoma-extra large; BIM: BCL2-like 11; CD11b: Integrin alpha M; CD11c: Integrin alpha X; CD19: Cluster of differentiation 19; CD3e: Cluster of differentiation 3 epsilon; CD4: Cluster of differentiation 4; CD45: Cluster of differentiation 45; CD8a: Cluster of differentiation 8 alpha; CTLA4: Cytotoxic T-lymphocyte-associated protein 4; F4/80: EGF-like module-containing mucin-like hormone receptor-like 1; Fibronectin: Fibronectin 1; gamma-H2AX: gamma-H2A histone family member X; GAPDH: Glyceraldehyde-3-phosphate dehydrogenase; GZMB: Granzyme B; Ki-67: Marker of proliferation Kiel 67; MHC II: Major histocompatibility complex class II; p21: Cyclin-dependent kinase inhibitor 1; p53: Tumor protein p53; PanCk: Pan-cytokeratin; PARP: Poly-(ADP-ribose) polymerase; PD-1: Programmed cell death protein 1; PD-L1: Programmed death ligand 1; Perforin: Perforin 1; S6: Ribosomal protein S6; SMA: Smooth muscle actin.

^2^As these IgGs do not cross-react and bind to mouse proteins, they were used as negative controls for downstream data normalization.

Suppl. Table S2. Protein fold changes in islet areas of interest. When comparing distal vs proximal areas, a negative fold change indicates that protein levels are higher in proximal areas, while a positive fold change indicates that protein levels are higher in distal areas. Similarly, when comparing proximal vs control areas, a negative fold change corresponds to protein levels that are higher in proximal areas, while a positive fold change indicates that protein levels are higher in control areas. Histone H3, S6 and GAPDH are housekeeping proteins used for normalization in the GeoMx assay.

| Protein* | Islet distal vs proximal fold change | Islet distal vs proximal adj. p-value | Islet proximal vs ctr fold change | Islet proximal vs ctr adj. p-value |
| --- | --- | --- | --- | --- |
| BAD | 1.03 | 0.65 | -1.02 | 0.95 |
| BCLXL | -1.08 | 0.39 | -1.11 | 0.68 |
| BIM | -1.07 | 0.82 | -1.22 | 0.20 |
| CD11b | 1.03 | 0.60 | -1.07 | 0.83 |
| CD11c | -1.13 | 0.28 | -1.53 | 0.01 |
| CD19 | 1.05 | 0.59 | 1.06 | 0.98 |
| CD3e | 1.05 | 0.47 | -1.04 | 0.98 |
| CD4 | -1.24 | 0.28 | -1.88 | 0.01 |
| CD45 | -1.69 | 0.00 | -2.56 | 0.01 |
| CD8a | -1.22 | 0.04 | -1.24 | 0.20 |
| Cleaved Caspase 3 | -1.13 | 0.82 | -1.13 | 0.69 |
| CTLA4 | 1.14 | 0.26 | 1.08 | 0.49 |
| F4/80 | 1.09 | 0.28 | -1.01 | 0.98 |
| Fibronectin | 1.05 | 0.41 | -1.04 | 0.69 |
| gamma-H2AX | 1.16 | 0.47 | 1.03 | 0.98 |
| GAPDH | 1.03 | 0.65 | -1.05 | 0.98 |
| GZMB | 1.28 | 0.01 | 1.18 | 0.37 |
| Histone H3 | -1.12 | 0.26 | -1.17 | 0.49 |
| Ki-67 | -1.00 | 0.82 | -1.09 | 0.83 |
| MHC II | -1.04 | 0.81 | -1.18 | 0.50 |
| p21 | -1.11 | 0.51 | -1.16 | 0.50 |
| p53 | 1.09 | 0.65 | -1.06 | 0.83 |
| PanCk | -1.02 | 0.88 | -1.14 | 0.37 |
| PARP | 1.12 | 0.26 | 1.06 | 0.98 |
| PD-1 | 1.03 | 0.57 | -1.12 | 0.68 |
| PD-L1 | 1.23 | 0.09 | 1.20 | 0.37 |
| Perforin | -1.05 | 0.76 | -1.34 | 0.28 |
| S6 | 1.26 | 0.01 | 1.27 | 0.28 |
| SMA | -1.18 | 0.11 | -1.12 | 0.94 |

*Abbreviations: BAD: BCL2-associated agonist of cell death; BCLXL: B-cell lymphoma-extra large; BIM: BCL2-like 11; CD11b: Integrin alpha M; CD11c: Integrin alpha X; CD19: Cluster of differentiation 19; CD3e: Cluster of differentiation 3 epsilon; CD4: Cluster of differentiation 4; CD45: Cluster of differentiation 45; CD8a: Cluster of differentiation 8 alpha; CTLA4: Cytotoxic T-lymphocyte-associated protein 4; F4/80: EGF-like module-containing mucin-like hormone receptor-like 1; Fibronectin: Fibronectin 1; gamma-H2AX: gamma-H2A histone family member X; GAPDH: Glyceraldehyde-3-phosphate dehydrogenase; GZMB: Granzyme B; Ki-67: Marker of proliferation Kiel 67; MHC II: Major histocompatibility complex class II; p21: Cyclin-dependent kinase inhibitor 1; p53: Tumor protein p53; PanCk: Pan-cytokeratin; PARP: Poly-(ADP-ribose) polymerase; PD-1: Programmed cell death protein 1; PD-L1: Programmed death ligand 1; Perforin: Perforin 1; S6: Ribosomal protein S6; SMA: Smooth muscle actin.

Suppl. Table S3. Comparison of protein levels in islet distal and ctr areas of interest. When comparing distal vs control areas, a negative fold change corresponds to protein levels that are higher in distal areas compared to control areas, while proteins with positive fold changes indicate that protein levels are higher in control areas. Histone H3, S6 and GAPDH are housekeeping proteins used for normalization in the GeoMx assay.

| Protein* | Islet distal vs ctr fold change | Islet distal vs ctr adj. p-value |
| --- | --- | --- |
| BAD | -1.05 | 0.74 |
| BCLXL | -1.03 | 0.82 |
| BIM | -1.14 | 0.63 |
| CD11b | -1.10 | 0.74 |
| CD11c | -1.35 | 0.33 |
| CD19 | 1.01 | 0.74 |
| CD3e | -1.09 | 0.74 |
| CD4 | -1.51 | 0.55 |
| CD45 | -1.51 | 0.55 |
| CD8a | -1.02 | 0.74 |
| Cleaved Caspase 3 | 1.00 | 0.82 |
| CTLA4 | -1.06 | 0.89 |
| F4/80 | -1.09 | 0.74 |
| Fibronectin | -1.09 | 0.55 |
| gamma-H2AX | -1.12 | 0.74 |
| GAPDH | -1.08 | 0.89 |
| GZMB | -1.08 | 0.94 |
| Histone H3 | -1.04 | 0.82 |
| Ki-67 | -1.08 | 0.74 |
| MHC II | -1.13 | 0.74 |
| p21 | -1.04 | 0.82 |
| p53 | -1.15 | 0.74 |
| PanCk | -1.11 | 0.61 |
| PARP | -1.06 | 0.74 |
| PD-1 | -1.15 | 0.74 |
| PD-L1 | -1.02 | 0.84 |
| Perforin | -1.28 | 0.63 |
| S6 | 1.01 | 0.84 |
| SMA | 1.06 | 0.74 |

*Abbreviations: BAD: BCL2-associated agonist of cell death; BCLXL: B-cell lymphoma-extra large; BIM: BCL2-like 11; CD11b: Integrin alpha M; CD11c: Integrin alpha X; CD19: Cluster of differentiation 19; CD3e: Cluster of differentiation 3 epsilon; CD4: Cluster of differentiation 4; CD45: Cluster of differentiation 45; CD8a: Cluster of differentiation 8 alpha; CTLA4: Cytotoxic T-lymphocyte-associated protein 4; F4/80: EGF-like module-containing mucin-like hormone receptor-like 1; Fibronectin: Fibronectin 1; gamma-H2AX: gamma-H2A histone family member X; GAPDH: Glyceraldehyde-3-phosphate dehydrogenase; GZMB: Granzyme B; Ki-67: Marker of proliferation Kiel 67; MHC II: Major histocompatibility complex class II; p21: Cyclin-dependent kinase inhibitor 1; p53: Tumor protein p53; PanCk: Pan-cytokeratin; PARP: Poly-(ADP-ribose) polymerase; PD-1: Programmed cell death protein 1; PD-L1: Programmed death ligand 1; Perforin: Perforin 1; S6: Ribosomal protein S6; SMA: Smooth muscle actin.

Suppl. Table S4. Protein fold changes in exocrine areas of interest. A negative fold change indicates that protein levels were higher in proximal areas, while a positive fold change indicates that protein levels were higher in distal areas. NB. Histone H3, S6 and GAPDH are housekeeping proteins that were used for normalization.

| Protein* | Fold change | Adjusted p-value |
| --- | --- | --- |
| BAD | 1.02 | 0.92 |
| BCLXL | -1.11 | 0.73 |
| BIM | -1.07 | 0.73 |
| CD11b | -1.02 | 0.92 |
| CD11c | -1.25 | 0.46 |
| CD19 | 1.16 | 0.28 |
| CD3e | -1.06 | 0.92 |
| CD4 | -2.44 | 0.01 |
| CD45 | -5.82 | 0.00 |
| CD8a | -1.27 | 0.20 |
| Cleaved Caspase 3 | 1.05 | 0.92 |
| CTLA4 | 1.07 | 0.92 |
| F4/80 | -1.10 | 0.92 |
| Fibronectin | -1.05 | 0.92 |
| gamma-H2AX | 1.27 | 0.30 |
| GAPDH | 1.02 | 0.92 |
| GZMB | 1.19 | 0.45 |
| Histone H3 | -1.52 | 0.01 |
| Ki-67 | -1.21 | 0.73 |
| MHC II | 1.08 | 0.45 |
| p21 | -1.05 | 0.92 |
| p53 | -1.08 | 0.92 |
| PanCk | 1.05 | 0.92 |
| PARP | -1.14 | 0.73 |
| PD-1 | 1.30 | 0.05 |
| PD-L1 | -1.02 | 0.92 |
| Perforin | 1.01 | 0.92 |
| S6 | 1.44 | 0.07 |
| SMA | -3.87 | 0.02 |

*Abbreviations: BAD: BCL2-associated agonist of cell death; BCLXL: B-cell lymphoma-extra large; BIM: BCL2-like 11; CD11b: Integrin alpha M; CD11c: Integrin alpha X; CD19: Cluster of differentiation 19; CD3e: Cluster of differentiation 3 epsilon; CD4: Cluster of differentiation 4; CD45: Cluster of differentiation 45; CD8a: Cluster of differentiation 8 alpha; CTLA4: Cytotoxic T-lymphocyte-associated protein 4; F4/80: EGF-like module-containing mucin-like hormone receptor-like 1; Fibronectin: Fibronectin 1; gamma-H2AX: gamma-H2A histone family member X; GAPDH: Glyceraldehyde-3-phosphate dehydrogenase; GZMB: Granzyme B; Ki-67: Marker of proliferation Kiel 67; MHC II: Major histocompatibility complex class II; p21: Cyclin-dependent kinase inhibitor 1; p53: Tumor protein p53; PanCk: Pan-cytokeratin; PARP: Poly-(ADP-ribose) polymerase; PD-1: Programmed cell death protein 1; PD-L1: Programmed death ligand 1; Perforin: Perforin 1; S6: Ribosomal protein S6; SMA: Smooth muscle actin.

Link to GitHub with additional supplementary material. Datasets and the R-code used to generate GeoMx and IHC analyses and figures can be found at https://github.com/claeslindhardt/IHCMousePancrea. Whole-slides scans of tissue sections can be found at Harvard Dataverse at https://dataverse.harvard.edu/dataverse/TekinetalSpatialNOD.
